# Integration of Transcriptome, Gross Morphology and Histopathology in the Gill of Sea Farmed Atlantic Salmon (*Salmo salar*): Lessons From Multi-Site Sampling

**DOI:** 10.3389/fgene.2020.00610

**Published:** 2020-06-19

**Authors:** Elżbieta Król, Patricia Noguera, Sophie Shaw, Eoin Costelloe, Karina Gajardo, Victoria Valdenegro, Ralph Bickerdike, Alex Douglas, Samuel A. M. Martin

**Affiliations:** ^1^School of Biological Sciences, Institute of Biological and Environmental Sciences, University of Aberdeen, Aberdeen, United Kingdom; ^2^Fish Health and Welfare, Marine Scotland Science, Aberdeen, United Kingdom; ^3^Centre for Genome-Enabled Biology and Medicine, University of Aberdeen, Aberdeen, United Kingdom; ^4^BioMar AS, Trondheim, Norway; ^5^Scottish Sea Farms, Stirling, United Kingdom

**Keywords:** proliferative gill disease, gene expression, RNA-seq, immune response, gill inflammation, aquaculture, climate change

## Abstract

The gill of teleost fish is a multifunctional organ involved in many physiological processes such as gas exchange, osmotic and ionic regulation, acid-base balance and excretion of nitrogenous waste. Due to its extensive interface with the environment, the gill plays a key role as a primary mucosal defense tissue against pathogens, as manifested by the presence of the gill-associated lymphoid tissue (GIALT). In recent years, the prevalence of multifactorial gill pathologies has increased significantly, causing substantial losses in Atlantic salmon aquaculture. The transition from healthy to unhealthy gill phenotypes and the progression of multifactorial gill pathologies, such as proliferative gill disease (PGD), proliferative gill inflammation (PGI) and complex gill disorder (CGD), are commonly characterized by epithelial hyperplasia, lamellar fusion and inflammation. Routine monitoring for PGD relies on visual inspection and non-invasive scoring of the gill tissue (gross morphology), coupled with histopathological examination of gill sections. To explore the underlying molecular events that are associated with the progression of PGD, we sampled Atlantic salmon from three different marine production sites in Scotland and examined the gill tissue at three different levels of organization: gross morphology with the use of PGD scores (macroscopic examination), whole transcriptome (gene expression by RNA-seq) and histopathology (microscopic examination). Our results strongly suggested that the changes in PGD scores of the gill tissue were not associated with the changes in gene expression or histopathology. In contrast, integration of the gill RNA-seq data with the gill histopathology enabled us to identify common gene expression patterns associated with multifactorial gill disease, independently from the origin of samples. We demonstrated that the gene expression patterns associated with multifactorial gill disease were dominated by two processes: a range of immune responses driven by pro-inflammatory cytokines and the events associated with tissue damage and repair, driven by caspases and angiogenin.

## Introduction

The gill of teleost fish is a multifunctional organ involved in many physiological processes such as gas exchange, osmotic and ionic regulation, acid-base balance and excretion of nitrogenous waste (Evans et al., 2005). To facilitate these functions, the gill tissue has evolved into a highly complex system of branching vascular structures (lamellae), separated from the external milieu only by a thin layer of gill epithelium and mucosa (Koppang et al., 2015; Salinas, 2015). The densely packed lamellar structure of the gill is highly advantageous because it provides a large surface area for oxygen transfer, amounting to approximately 0.1–0.4 m^2^ of lamellar surface per kg of body mass (Maina, 2011; Park et al., 2014). However, having such an extensive interface with the environment comes at a high price (reviewed in Nilsson, 2007). Firstly, the large respiratory surface area may contribute to the increased water and ion fluxes that need to be counteracted by energetically expensive ion pumping. That so-called osmorespiratory compromise, which is a trade-off between high gill permeability (to promote respiratory gas exchange) and low gill permeability (to limit unfavorable water and ion fluxes), has been demonstrated in freshwater fish during exercise and in hypoxia-intolerant species (e.g., salmonids) during the periods of exposure to low-oxygen water (Onukwufor and Wood, 2018; Morgenroth et al., 2019). Secondly, the highly complex vasculature of the gill is prone to mechanical injuries, increasing the risk of hemorrhage. Because the gill is the only organ in fish that receives the entire cardiac output, large or frequent gill hemorrhages can be fatal. Thirdly, the large lamellar surface area may facilitate the uptake of toxic substances, both those that occur naturally (such as ammonia, algal toxins and metal ions) as well as man-made pollutants (e.g., pesticides, herbicides, fertilizers, detergents, industrial chemicals and microplastics) (McIntyre et al., 2018; Kintner and Brierley, 2019). Finally, the close proximity of blood and environmental water across the large respiratory surface area provides (1) major ports of entry for pathogens via transepithelial transport and (2) attachment sites for water-born parasites. To combat pathogens and parasites, the gill is equipped with the gill-associated lymphoid tissue (GIALT), adding another dimension to the importance of this organ to fish functioning and survival (Koppang et al., 2015; Salinas, 2015; Xu et al., 2020).

To avoid ‘the large interface’ penalties, fish constantly adjust the functional size of the gill tissue to match their current oxygen demands (Nilsson, 2007). When faced with changes in their need for oxygen uptake (due to changes in metabolic rate or water oxygen content), fish are known to (1) regulate the water flow over the gill by adjusting the volume and frequency of buccal pumping and/or (2) regulate the blood flow inside the gill to alter the perfusion levels of lamellae. New evidence suggests that many teleost species may also undergo extensive gill tissue remodeling, during which lamellae are either completely embedded in a cell mass (to minimize the respiratory surface area) or fully protruded (to maximize the functional size of the tissue), with many potential intermediate stages between these extremes (Sollid et al., 2003; Anttila et al., 2015). Whether these regulatory and compensatory mechanisms aiming to optimize gill performance are sufficient for fish to cope with climate change, especially in the areas of high human impacts, remains unknown.

Fish are predominantly ectothermic and any increases in water temperature resulting from climate change will increase their metabolic rate and thus demands for oxygen. The Q_10_ for metabolism in fish (the fold increase in metabolic rate with a 10% increase in water temperature) varies from 1.5 to 2 (White et al., 2006). But the elevated oxygen demand is not the only challenge that the gill tissue is facing. Warmer water holds less oxygen and rising global temperatures alter oceanic circulation, which altogether may decrease the oxygen supply available for fish to support their increased metabolism (McBryan et al., 2013). It has been estimated that the open ocean lost ∼2% (77 billion metric tons) of its oxygen over the past 50 years (reviewed in Breitburg et al., 2018). The extent of deoxygenation of coastal waters may even be greater, due to eutrophication from agriculture and sewage runoff (Diaz and Rosenberg, 2008). Furthermore, environmental changes, including temperature increases, have been linked to enhanced expression of marine infectious diseases (Burge et al., 2014). Many marine organisms, including marine pathogens, are shifting their distributions poleward or to deeper waters as ocean temperatures warm (Nye et al., 2009; Pinsky et al., 2013). As a result, many species of fish are at risk of being exposed to novel pathogens and parasites that were not part of their evolutionary history (LeBlanc et al., 2019). Climate change may also affect the fish-pathogen interactions that are already well established, for example by altering pathogen virulence, overall pathogenicity and/or host immune response (Benedicenti et al., 2019; Laurin et al., 2019). Given the fact that in fish most environmental stressors converge at the level of the gill, performance of this tissue and its capacity to accommodate environmental change are central to understand short- and long-term impacts of warming and hypoxia on fish species (Akbarzadeh et al., 2018; McCormick and Regish, 2018; Houde et al., 2019). Monitoring gill health is especially important in farmed marine fish, which are restricted to coastal waters and have limited microhabitat choice.

In recent years, the prevalence of gill damage and disease in farmed marine fish has significantly increased, leading to substantial losses in the Scottish and global Atlantic salmon (*Salmo salar*) aquaculture industry (Young et al., 2007; Shinn et al., 2015; Nowak and Archibald, 2018). Although gill-related mortality varies from site to site and between years, July to December in Northern Hemisphere is considered the higher risk period because the sea during these months is warmer. With the projected increase in sea water temperature associated with climate change, gill pathologies are now regarded as the greatest challenge to the farmed salmon sector due to direct and indirect impacts on productivity. Losses may occur through direct mortalities, poor growth rates linked to decreased feed efficiency and because of the increased risk of co-infection (Rodger, 2007; Downes et al., 2018). The transition from healthy to unhealthy gill tissue happens through the progression of various gill pathologies such as amoebic gill disease (AGD), proliferative gill disease (PGD), proliferative gill inflammation (PGI) and complex gill disorder (CGD), which are commonly characterized by epithelial hyperplasia, lamellar fusion and inflammation (Steinum et al., 2010; Matthews et al., 2013; Mitchell et al., 2013; Herrero et al., 2018; Gjessing et al., 2019). The diagnostic of gill pathologies is often difficult because single-cause diseases (such as AGD) are compounded by multi-cause (multifactorial) disorders such as PGD, PGI or CGD, with complex etiology and potentially synergistic effects of co-infections (Gjessing et al., 2017; Gunnarsson et al., 2017; English et al., 2019). Due to overlapping symptoms of multifactorial gill inflammation, the terms PGD, PGI and CGD are often used interchangeably, with the first two historically referring to salmon farmed in Scotland (PGD) and Norway (PGI), while the last one highlighting the growing complexity of gill diseases and encompassing the syndromes referred to as PGD and PGI (Rozas-Serri, 2019). The multifactorial gill diseases are associated with a number of causative agents, including phytoplankton, zooplankton, viruses, fungi, bacteria and larger parasites (Gjessing et al., 2017; Herrero et al., 2018), with climate change contributing to emerging new aquaculture pathogens (Bayliss et al., 2017). Furthermore, gill diseases not only directly affect fish health and performance, but also creates a challenge for farmed salmon producers as they are required to treat compromised fish, increasing the risk of mechanical injuries to the gill vasculature (Powell et al., 2005). To meet the challenge of salmon gill health, the sector needs non-invasive monitoring tools for (1) early detection of gill pathologies, (2) diagnostics that is sufficient to establish appropriate treatment and (3) evaluation of effectiveness of treatment and recovery of gill function. It is important that such initiatives target multifactorial diseases, as they are becoming progressively more prevalent (Rahel and Olden, 2008).

The current practice is to regularly check sea farmed salmon for any signs of gill inflammation by *in situ* gross morphology examination of gill arches, using the PGD scoring system from 0 (no macroscopic pathology) to 5 (severe macroscopic pathology) (Gill Health Initiative, 2015; Bloecher et al., 2018). This is a non-invasive procedure that requires only light anesthesia and can be performed on a weekly basis in fish from across a facility. However, the applicability of the PGD scoring system to grade inflammation has never been tested in a systematic way. Because PGD is a multifactorial disease, progression from low to high PGD scores is likely to have a large component of case specificity, depending on the combination of pathogens involved, the overall health status of the fish and the environmental conditions. Despite complex etiology, the phenotypes generated by PGD are macroscopically similar (inflammation and hyperplasia of respiratory epithelium), which suggests that at least some of the underlying mechanisms associated with this pathology may be common (Mitchell and Rodger, 2011; Bruno et al., 2013). Unlocking these shared mechanisms is essential for early detection of gill disease, developing strategies to improve gill health and for fine-tuning salmon husbandry practices to the challenging conditions of a rapidly changing marine environment.

To identify the common gene expression patterns associated with gill inflammation, we examined Atlantic salmon from three marine production sites in Scotland. The sites and time of sampling were chosen to ensure (1) differences in fish cohort (hatchery origin, on-site treatments and overall health status), (2) diversity of pathogen and (3) differences in local environment. For each site, we selected fish with low and high PGD scores, analyzed their gill transcriptome (RNA-seq) and gill histopathology (microscopic examination), and then integrated these data to explore gene expression patterns associated with multifactorial gill disease.

## Materials and Methods

### Sampling and Gross Morphology

Fish (Atlantic salmon, *Salmo salar*) were sampled at three marine production sites belonging to Scottish Sea Farms (A on Isle of Mull and B and C in Shetland) between October 2017 and March 2018 (for details see Table 1 and Supplementary Figure 1). All fish were of strain Fanad and originated from the same egg fertilization batch. They were reared in different hatcheries (Couldoran, Pettigo-Damph and Knock-Frisa for sites A, B and C, respectively) for one year and entered the sea in spring 2017.

**TABLE 1:** Sampling details, fish metrics and background.

Site	Date	SWT (°C)^1^	Number of fish	Body mass (kg)^2^	Body length (cm)^2^	*K*^2,3^
A (Isle of Mull)	19 Oct 2017	13.4	31	2.4 ± 0.5	55 ± 3	1.4 ± 0.1
B (Shetland)	24 Nov 2017	13.9	20	2.4 ± 0.4	55 ± 3	1.4 ± 0.2
C (Shetland)	22 March 2018	5.7	25	2.6 ± 0.7	55 ± 4	1.5 ± 0.2

*^1^Sea water temperature at 5 m depth; ^2^Values are means ± standard deviations; ^3^Fulton’s condition factor (K) calculated as 100 × body mass in g/body length in cm^∧^3.*

**FIGURE 1:**
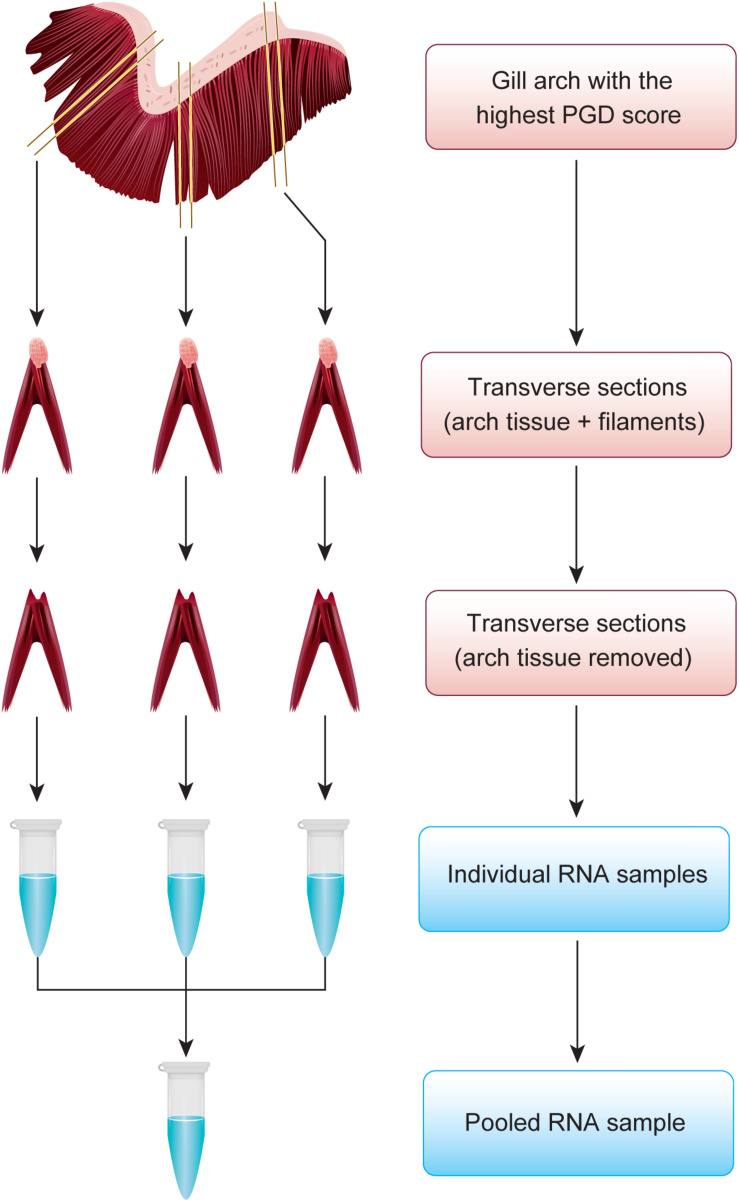
Approach to representatively divide the gill arch with the highest PGD score between transcriptome profiling and histopathological examination. For transcriptomic profiling, 3 transverse sections from dorsal, medial and ventral regions of the gill were subjected to total RNA extraction to generate individual RNA samples, which were subsequently pooled. All remaining tissue was used for histopathology.

During sampling, randomly selected fish (76 in total) were placed in an anesthetic bath (∼20 g of MS-222/150 L) for 5–10 min, followed by macroscopic examination (gross morphology) of gill tissue as per routine procedure to monitor fish welfare indicators. This procedure is performed by fish health professionals, using semi-quantitative 6-grade scoring systems for PGD, from 0 (no visual pathology) to 5 (severe visual pathology) (Gill Health Initiative, 2015; Bloecher et al., 2018). Each of the 8 gill arches (4 on left and 4 on right side) was scored separately, generating in total 8 PGD scores per fish. Although scoring for PGD is typically non-destructive and requires only light anesthesia (e.g., 10 g of MS-222/150 L), fish in our study were subjected to terminal anesthesia for subsequent harvesting of tissue samples. Immediately after PGD scoring, fish were bled and the gill arch with the highest PGD score was excised for both transcriptome profiling and histopathological examination. For transcriptomic profiling, 3 transverse sections (gill arch ∼3 mm long with 3–4 pairs of filaments) from dorsal, medial and ventral regions of the gill (Figure 1) were transferred to RNAlater^®^ (Sigma-Aldrich, St. Louis, MO, United States), kept at 4°C overnight for equilibration and then stored at −80°C prior to RNA extraction. All remaining tissue from the same gill arch was transferred to freshly prepared seawater Davidson’s fixative (Cadoret et al., 2013) for 24 h, followed by a short-term exposure to 10% neutral buffered formalin before tissue processing for histopathological examination.

### Selection of Fish With Low and High PGD Scores

Grouping all 76 sampled fish by their highest PGD score and site demonstrated that each site had fish with PGD scores 1, 2 or 3, but not 0 or 5, and only rarely 4 (Table 2). Thus, the comparison between fish with low and high PGD scores was restricted to PGD scores 1 and 3, respectively. In total, 45 gill arches (27 with PGD score 1 and 18 with PGD score 3) were subjected to total RNA extraction, but the final RNA-seq sample size dropped to 43 gill arches (from 43 fish), due to losses at the both pre- and post-sequencing stage (Table 2).

**TABLE 2:** Results of gross gill scoring for proliferative gill disease (PGD), performed on all 8 gill arches of individual fish to identify the gill arch with the highest PGD score for each fish.

		Number of fish grouped by their highest PGD score^2^					
Site	Median PGD score^1^	Score 0	Score 1	Score 2	Score 3	Score 4	Score 5
A (Isle of Mull)	1 (range 1–3.5, n = 31)	-	**10**	10	**10**	1	-
B (Shetland)	1 (range 1–2, n = 20)	-	**8**^3^	9	**3**	-	-
C (Shetland)	1 (range 0–3, n = 25)	-	**9**	10	**5**^4^	1	-

*The gill arches (one from each fish) subjected to transcriptome and histopathological examination (PGD scores 1 and 3) are presented in bold. ^1^Values are median PGD scores per fish (calculated from all 8 gill arches) per site (calculated for n fish), with range referring to fish with minimal and maximal median PGD scores at each site; ^2^- denotes no fish; ^3^Final n = 7, because one total RNA sample was degraded; ^4^Final n = 4, because one RNA-seq sample was identified as an outlier and removed from analysis.*

### RNA Extraction

Total RNA extraction was performed on individual gill transverse sections (45 gills × 3 sections) after removing the arch tissue and leaving only full-length filaments for further processing (Figure 1). Briefly, the RNA was isolated by homogenization of ∼100 mg of gill tissue in TRIzol^®^ Reagent (Ambion by Life Technologies, Carlsbad, CA, United States), using 3 mm tungsten carbide beads and a TissueLyser II Disruption System (Qiagen GmbH, Hilden, Germany). Following isolation, the RNA was quantified by spectrophotometry (NanoDrop Technologies, Wilmington, DE, United States) and its integrity was confirmed by electrophoresis (Agilent Technologies, Santa Clara, CA, United States). The three individual RNA samples that originated from the same gill were then pooled to generate a single RNA sample per gill tissue (*n* = 45 RNA pools in total), with an equimolar contribution of RNA from dorsal, medial and ventral regions of the gill to each pool. All but one pooled gill RNA samples had a 260/280 ratio > 1.8 and RIN number > 9.3, thus meeting the criteria for RNA-sequencing. The sample with degraded RNA was eliminated from further processing.

### RNA-seq Library Preparation and Sequencing

RNA-seq library preparation and sequencing were carried out by Edinburgh Genomics at the University of Edinburgh (United Kingdom). The libraries for each of the 44 samples were constructed using the TruSeq Stranded mRNA Sample Preparation Kit (Illumina, San Diego, CA, United States), according to the manufacturer’s instructions. The paired-end sequencing (50 bp from each end) was performed on the NovaSeq 6000 system with S2 flow cell (Illumina, San Diego, CA, United States) at a sequencing depth of ∼50 million read pairs per library. The raw reads in BCL format were converted to FastQ format with bcl2fastq2 Conversion Software v2.19.1 (Illumina, San Diego, CA, United States). All raw sequences have been deposited in the ArrayExpress repository^1^ under accession number E-MTAB-8855.

### Read Mapping

To assess the quality of the sequencing data, reads were analyzed with FastQC v0.11.8 (Andrews, 2010). Sequencing adaptors and sequences shorter than 20 bp were removed using Flexbar v3.4.0 (Dodt et al., 2012). Filtered reads were then mapped to the Atlantic salmon reference genome ICSASG_v2 (GenBank: GCF_000233375.1, Lien et al., 2016) using HISAT2 v2.1.0. (Kim et al., 2015) with the stranded library preparation parameter. Overall, alignment rates ranged from 93.2 to 95.7%. Aligned reads were counted at gene locations using featureCounts v1.6.4 (Liao et al., 2014). For multi-mapping reads, a fractional count (1/n) was generated for each reported alignment of the multi-mapping read, with n reflecting the total number of alignments reported for that read.

### Differential Gene Expression

Differential expression analysis was performed using the Bioconductor package edgeR v3.22.5 (Robinson et al., 2010) in R v3.5.1 (R Core Team, 2018). Genes with a CPM (count per million) < 1 in three or more samples were removed, resulting in 35996 genes for analysis. Filtered counts were subsequently normalized using a trimmed mean of *M*-values (TMM) between each pair of samples. Based on exploratory data analysis, one library was identified as an outlier and removed from subsequent analysis (for details on sample size see Table 2). Data for the remaining 43 fish were modeled using a negative binomial generalized log-linear mixed model that included both group (PGD scores 1 and 3) and site (A, B and C) as fixed effects. In total, four contrasts were generated from the same model: PGD 3 vs PGD 1 (with the site effect blocked) and then sites A vs C, B vs C and A vs B (with the group effect blocked). Differentially expressed genes (DEGs) were identified at false discovery rate (FDR) < 0.01 and absolute Log_2_ fold change (FC) > 1.

### Functional Analysis of Gene Expression

Salmon DEGs were mapped to human orthologs to generate HGNC (HUGO Gene Nomenclature Committee) gene identifiers for functional analysis of the RNA-seq results. This approach has been demonstrated to improve biological interpretation of the salmon gene expression profiles by providing access to well-annotated databases and tools for mammalian model organisms, despite limitations of the mapping due to the extra genome duplication events in teleost fish and species-specific differences in gene function and molecular pathways (Song et al., 2014; Król et al., 2016). Mapping was done by aligning salmon transcript sequences to the protein sequences from the human genome (release 88, downloaded from Ensembl at^2^) using BLASTX (version 2.2.31) (Camacho et al., 2009) with an E-value cut off of 0.00001 and a maximum of one target sequence for every transcript. As one transcript can have multiple hits against one target sequence, custom Python scripts were used to filter the blast results to contain only the hit with the highest identity for each transcript. Although the majority of the salmon genes mapped to a unique human ortholog, some salmon genes mapped to the same human ortholog (for details see Results). To obtain a single expression value (mean Log_2_ FC) per HGNC gene identifier, the expression of the salmon genes mapped to the same human ortholog was either averaged (if contributing salmon genes had similar expression profiles) or based on the expression of the salmon gene that was more abundant (if contributing salmon genes had contrasting expression profiles). For biological interpretation of the RNA-seq results, human orthologs of the salmon DEGs along with their Log_2_ FC values were analyzed using Ingenuity Pathway Analysis (IPA, QIAGEN Redwood City, www.qiagen.com/ingenuity) to explore (1) enrichment of canonical pathways, (2) upstream regulators and (3) downstream effects associated with these genes. The same set of genes was also submitted to PANTHER Classification System (Mi et al., 2013) to perform Gene Ontology (GO) enrichment analysis.

### Gill Histopathology

Gill tissue was routinely dehydrated in ethanol, equilibrated in xylene and embedded in paraffin wax according to standard histological techniques (Bancroft and Gamble, 2007). Sagittal sections (3 μm) of the gill arch were cut with a microtome and mounted onto microscope slides. These sections were then subjected to haematoxylin and eosin (H&E) staining. All sections were digitized at 40 × magnification, using the Olympus dotSlide 2.1 Virtual Slide System (Olympus Corporation, Tokyo, Japan). The resultant images were randomized to ensure blinded examination and then scored using a system developed to assess gill histopathology in sea farmed Atlantic salmon (Mitchell et al., 2012), with minor amendments. The details of the scoring system are presented in Table 3.

**TABLE 3:** Semi-quantitative scoring system used for histopathological examination of gill tissue in sea farmed Atlantic salmon (adapted and modified from Mitchell et al., 2012).

Parameter	Description
**Index criteria with scores 0–3^1^**	
LH	Lamellar hyperplasia
LF	Lamellar fusion
LO	Lamellar oedema
CA	Cellular anomalies
**Ancillary criteria with scores 0–3^1^**	
In	Inflammation (presence of inflammatory cells outside blood vessels)
Eg	Eosinophilic granular cells (numbers higher than normal in gill filaments)
Cc	Chloride cells (numbers higher than normal and/or abnormal location)
Cd	Circulatory disturbances (thrombi, telangiectasis, congestion)
Ib	Interlamellar blood (hemorrhage)
Ch	Cellular hypertrophy (hydropic degeneration of lamellar cells)
bE	*Epitheliocystis*-like bacteria
bT	*Tenacibaculum*-like bacteria (mats of filamentous bacteria on lamellar surfaces)
Ob	Other bacteria
Pp	Protist parasites, *Neoparamoeba*-like
Op	Other parasites or agents

*^1^Scoring system 0–3, with 0 = no pathological changes, 1 = mild changes affecting < 10% of gill tissue, 2 = moderate changes affecting 10–50% of gill tissue, and 3 = severe changes affecting > 50% of gill tissue.*

Prior to the analysis, three variables were excluded (bE, bT and ob) as scores were invariant across fish (see Results). To compare the gill histopathology scores between PGD groups (1 and 3) and also between fish from different sites (A, B and C), non-metric multidimensional scaling (NMDS) was performed using the metaMDS function from the vegan package (Oksanen et al., 2019) in R. For the NMDS analysis, a Jaccards dissimilarity index was used to calculate the dissimilarity matrix, two dimensions were specified and 100 random starts used. Similarities between fish histopathology scores were visualized using a biplot with 95% confidence ellipses around the group centroids and variable vectors included.

## Results

### Phenotypic Characteristics of Fish

**FIGURE 2:**
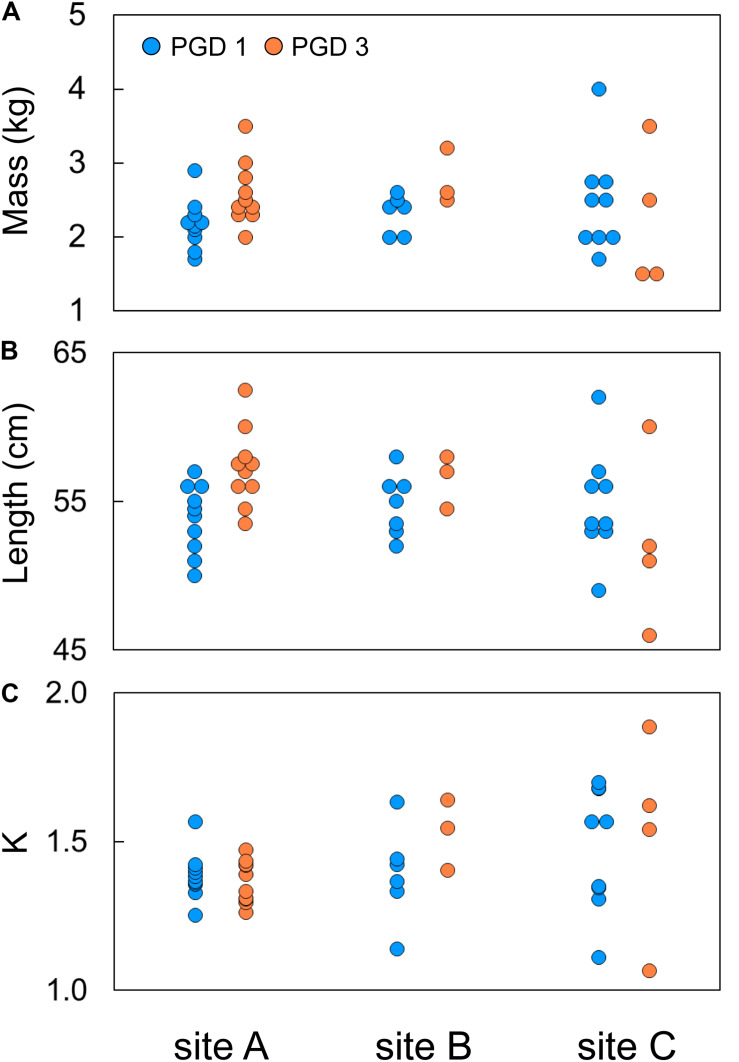
Individual body mass **(A)**, body length **(B)** and Fulton’s condition factor K **(C)** of 43 fish subjected to RNA-seq experiment, plotted by group (PGD 1 and PGD 3) and site (A, B and C). For formula to calculate K, see Table 1.

Body mass of 43 fish subjected to the RNA-seq experiment varied from 1.5 to 4.0 kg, with their body length ranging from 47 to 62 cm (Figures 2A,B). The effects of group (PGD 1 and PGD 3) and site (A, B and C) on body mass and length were not significant (*P* > 0.05, 2-way ANOVA). As a result, the fish did not differ in their Fulton’s condition factor K (*P* > 0.05, 2-way ANOVA), which varied from 1.1 to 1.7 (Figure 2C). Given the small sample size (Table 2), these results should be treated with caution.

### Gill Transcriptome

**FIGURE 3:**
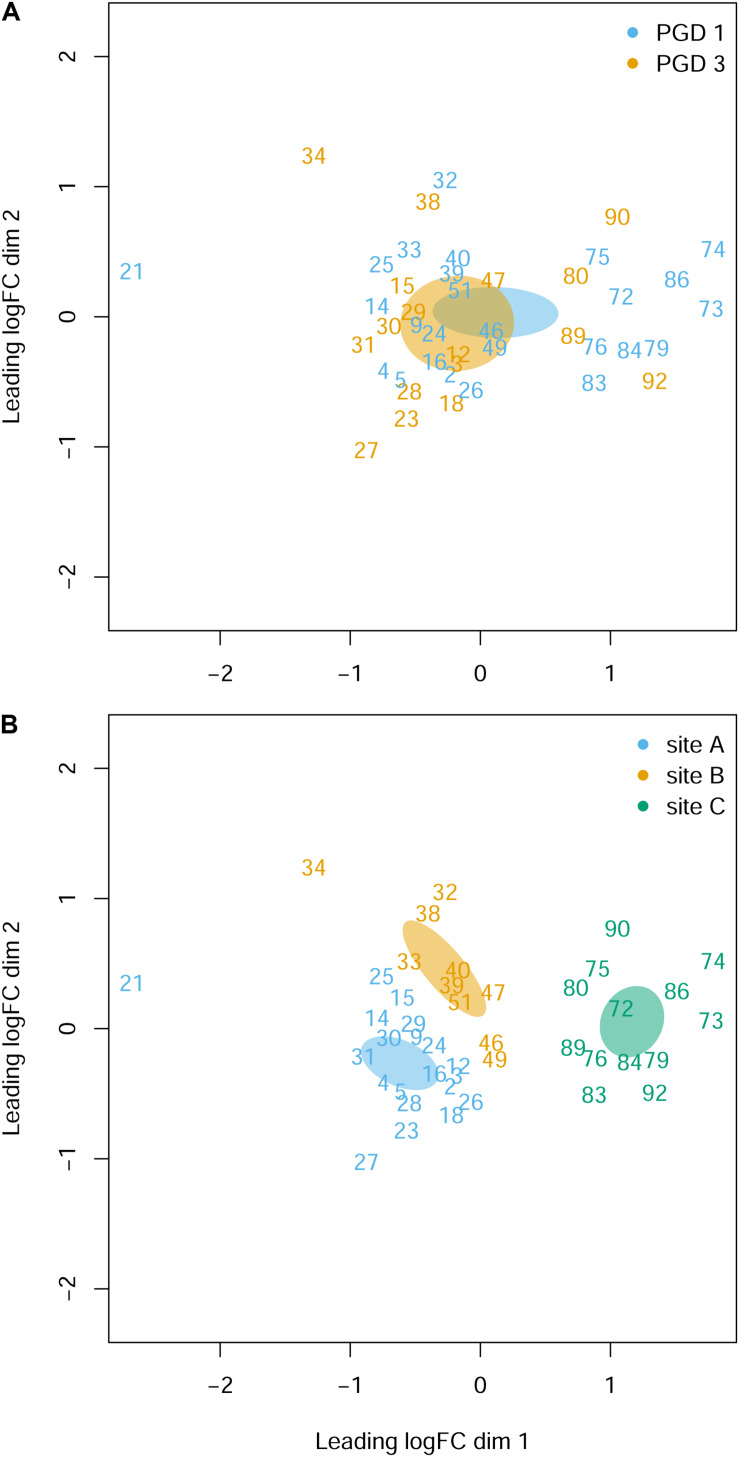
Gill transcriptome of 43 fish grouped by PGD score **(A)** and site **(B)**. Each panel shows a NMDS plot of gene expression profiles between different fish (numbers are fish IDs). The distances on the plots correspond to the leading fold change (FC), which is the average (root-mean-square) Log_2_ FC for the 500 genes most divergent between each pair of fish. Ellipses indicate 95% confidence intervals, overlapping for fish grouped by PGD score **(A)** but not for fish grouped by site **(B)**. The stress value of the NMDS ordination is 0.162.

The NMDS analysis of 43 gill transcriptomes revealed no differences between 26 fish with PGD score 1 and 17 fish with PGD score 3, as indicated by their overlapping 95% confidence intervals (Figure 3A). This finding was reinforced by differential gene expression analysis, which showed 0 DEGs for comparison PGD 3 vs PGD 1 (Table 4 and Supplementary Table 1). In contrast, gill transcriptomes were clearly separated by site, with no overlap in 95% confidence intervals for 20 fish from site A, 10 fish from site B and 13 fish from site C (Figure 3B). The site-specific differences in the gill transcriptomes are reflected in the number of DEGs identified between the sites: 1360 for A vs C, 708 for B vs C and 240 for A vs B (Table 4, for the lists of DEGs see Supplementary Tables 2–4).

**TABLE 4:** Results of differential gene expression analysis performed on gill transcriptome of 43 fish to elucidate the differences between groups (PGD 3 vs PGD 1) and sites (A vs C, B vs C and A vs B).

		Number of differentially expressed genes (DEGs)		
Comparison	N^1^	Total	Upregulated	Downregulated
PGD 3 vs PGD 1	17 vs 26	0	0	0
sites A vs C	20 vs 13	1360	858	502
sites B vs C	10 vs 13	708	492	216
sites A vs B	20 vs 10	240	124	116

*Genes were considered differentially expressed at FDR < 0.01 and absolute Log_2_ FC > 1. ^1^Number of fish used for each comparison.*

### Gill Histopathology

**FIGURE 4:**
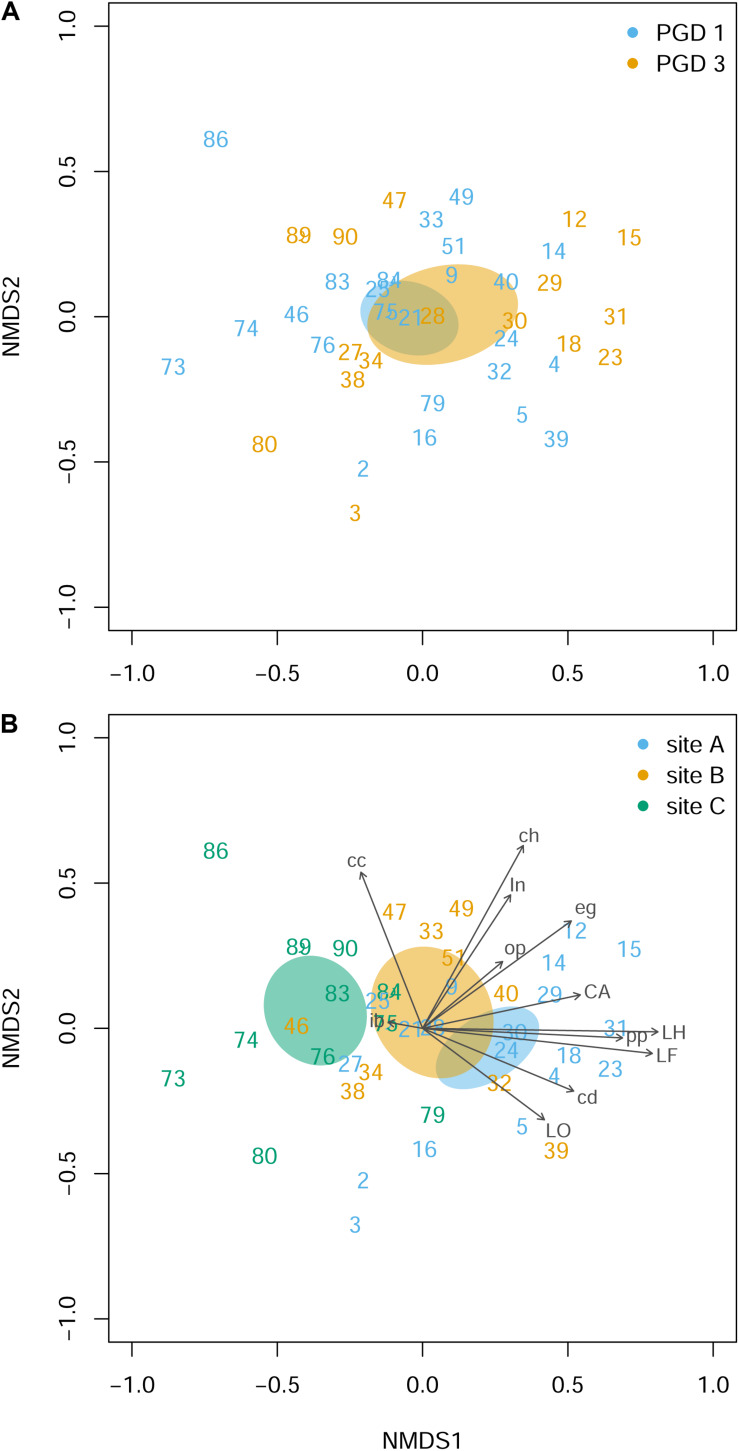
Gill histopathology of 43 fish grouped by PGD score **(A)** and site **(B)**. Each panel shows a NMDS plot of histopathological profiles between different fish (numbers are fish IDs). The distances on the plots are calculated from the scores of 12 histopathological parameters (LH, LF, LO, CA, in, eg, cc, cd, ib, ch, pp and op), with 3 parameters (bE, bT and ob) removed from the analysis because all scores were 0 (for parameter abbreviations and details see Table 3 and Supplementary Figure 2). Ellipses indicate 95% confidence intervals, overlapping for fish grouped by PGD score **(A)** and for fish from sites A and B but not from C **(B)**. Vectors are fitted to visualize the contribution of histopathological parameters to the ordination. The stress value of the NMDS ordination 0.190.

Histopathological examination of the gill tissue generated 15 scores (ranging from 0 to 3) per fish, focusing on 4 index and 11 ancillary criteria (Supplementary Table 5). Distribution of the histopathological scores by PGD score (1 and 3) and site (A, B and C) for each of 15 criteria is presented in Supplementary Figure 2. The NMDS analysis of the gill histopathology results for 43 fish showed no differences between 26 fish with PGD score 1 and 17 fish with PGD score 3, as indicated by their overlapping 95% confidence intervals (Figure 4A). In contrast, gill histopathology differed between sites, with 1) fish from site C being clearly separated from other fish and 2) fish from site A overlapping with fish from site B (Figure 4B). The position of the site-associated clusters along the NMDS1 axis indicates that fish from site C (with lower scores) show relatively low level of gill histopathology, while fish from sites A and B (with higher scores) have gill tissue with relatively moderate changes in histopathology. The main drivers of these differences are lamellar hyperplasia (LH), lamellar fusion (LF), cellular anomalies (CA) and *Neoparamoeba*-like protist parasites (pp), as evidenced by the vectors paralleled to the NMDS1 axis in Figure 4B. Overall, fish from site A and independently from site B developed significantly higher grade inflammation than fish from site C, thus facilitating the search for the molecular bases of multifactorial gill disease.

### Identification of Gene Expression Patterns Associated With Multifactorial Gill Disease

**FIGURE 5:**
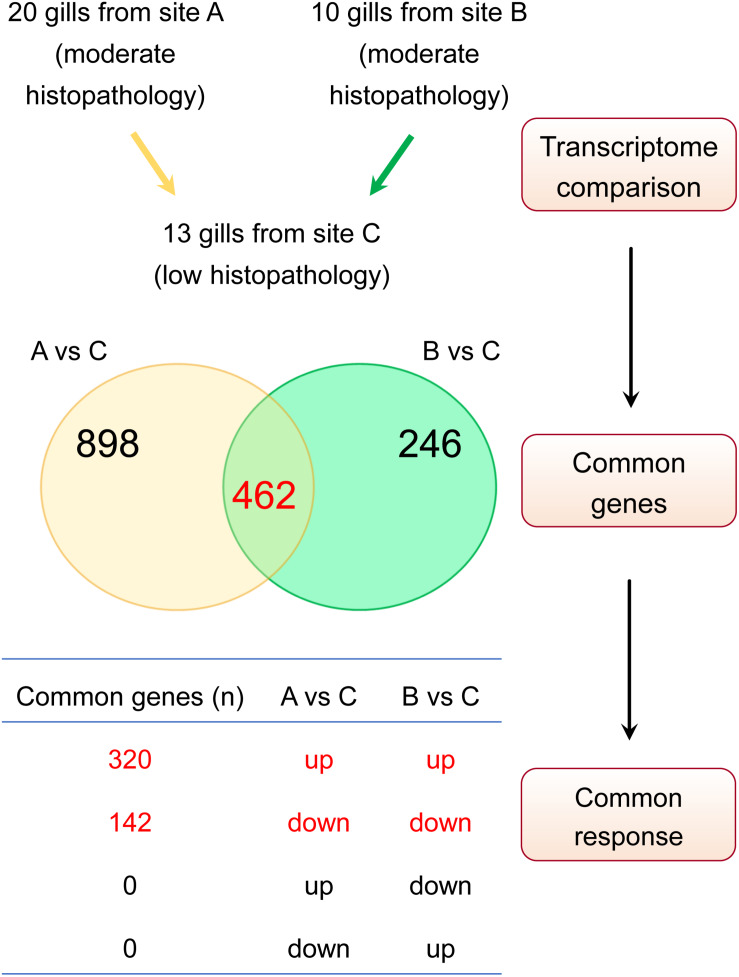
Approach to identify gene expression patterns associated with multifactorial gill disease. Based on the histopathological examination, A and B were classified as two independent sites with relatively moderate gill histopathology, while C was classified as a site with relatively low gill histopathology. Firstly, the lists of differentially expressed genes (DEGs) for A vs C and B vs C contrasts were generated (FDR < 0.01 and absolute Log_2_ FC > 1), yielding 1360 and 708 transcripts, respectively. Secondly, both lists of DEGs were checked for common genes (462 in total). Finally, the list of common genes was screened for a commonality of response to ensure that only genes either upregulated or downregulated in both sets of DEGs were considered to constitute a common gene expression profile of non-specific gill inflammation.

Our approach to identify the common gene expression patterns of non-specific gill inflammation is explained in Figure 5. Specifically, all gill transcriptomes from sites A and B (with moderate gill histopathology) were independently compared to all gill transcriptomes from site C (with low gill histopathology), which resulted in 1360 and 708 DEGs (at FDR < 0.01 and absolute Log_2_ FC > 1) for A vs C and B vs C contrasts, respectively (Table 4, for the lists of DEGs see Supplementary Tables 2, 3). A relatively large number of genes were common between these two comparisons (462 DEGs in total), including 354 protein-coding genes, 35 immunoglobulin gene segments, 15 pseudogenes and 58 non-coding RNAs (Table 5, for the list of common DEGs see Supplementary Table 6). The Log_2_ FC values for these genes were averaged between A vs C and B vs C contrasts to provide one expression value per common gene. Importantly, all common DEGs show the same direction of change in both the moderate-to-low histopathology contrasts, i.e., they are either upregulated or downregulated in relation to site C, as indicated in Figure 5. That commonality of the response suggests that the identified DEGs are part of the gene expression profile indicative of progression from lower to higher grade inflammation of gill tissue. To understand the underlying mechanisms, we focused on the 354 protein-coding DEGs and predicted their functionality through mapping to the human orthologs.

**TABLE 5:** Characterisation of 462 potential gene expression markers associated with gill disease.

	Number of genes			
Gene type	Total	HGNC +	HGNC −	IPA/GO
Protein-coding	354	311	43	311 → 235
Immunoglobulin gene segments	35	0	35	0
Pseudogenes	15	1	14	0
Non-coding RNA	58	23	35	0

*Mapping to human orthologs (BLASTX, E-value < 0.00001, top hit) generated in total 334 salmon genes with HGNC gene identifiers (HGNC +) and 128 salmon genes with no human orthologs (HGNC −). Only 311 protein-coding salmon genes with 235 unique HGNC gene identifiers were subjected to functional analysis of gene expression (IPA/GO). Abbreviations: HGNC, HUGO Gene Nomenclature Committee; IPA, Ingenuity Pathway Analysis; GO, Gene Ontology.*

### Mapping Salmon Genes to Human Orthologs

The results of blasting all 462 salmon DEGs associated with multifactorial gill disease against the protein sequences from the human genome (BLASTX, *E*-value < 0.00001, top hit) are presented in Table 5 and Supplementary Table 6. Among them, 311 of 354 protein-coding salmon genes were matched to HGNC gene identifiers, but not all of them were unique. Specifically, 191 protein-coding salmon transcripts mapped uniquely to one human ortholog, while the remaining 120 protein-coding salmon transcripts mapped to 44 human orthologs, with > 1 salmon gene mapping to the same HGNC gene identifier (Supplementary Table 7). The expression of salmon genes mapped to the same human ortholog was averaged to provide one expression value (mean Log_2_ FC) per HGNC gene identifier, if the response of the salmon genes was consistent (i.e., all contributing salmon genes were either upregulated or downregulated, but not both). In two cases with the contrasting expression of salmon genes (PRF1 and CTSV, for details see Supplementary Table 7), the mean Log_2_ FC was based on the expression of the salmon gene that was more abundant (higher CPM). As a result, the expression patterns of 311 protein-coding salmon DEGs were represented by 235 human orthologs and their corresponding 235 expression values (Log_2_ FC for 191 unique mapping and mean Log_2_ FC for 44 multiple mapping), which were then used to predict functionality.

### Functional Analysis of Gene Expression Patterns Associated With Multifactorial Gill Disease

Biological interpretation of the transcriptomic changes in gills with moderate histopathology (sites A and B) vs low histopathology (site C) was performed on the expression profiles of 311 protein-coding salmon DEGs converted into 235 human orthologs (Supplementary Table 7), using (1) IPA for enrichment of canonical pathways, upstream regulators and downstream effects and (2) PANTHER Classification System for enrichment of GO terms (Biological Process). The predictions made by IPA also require the expression values, while GO enrichment analysis is based on the list of DEGs.

**FIGURE 6:**
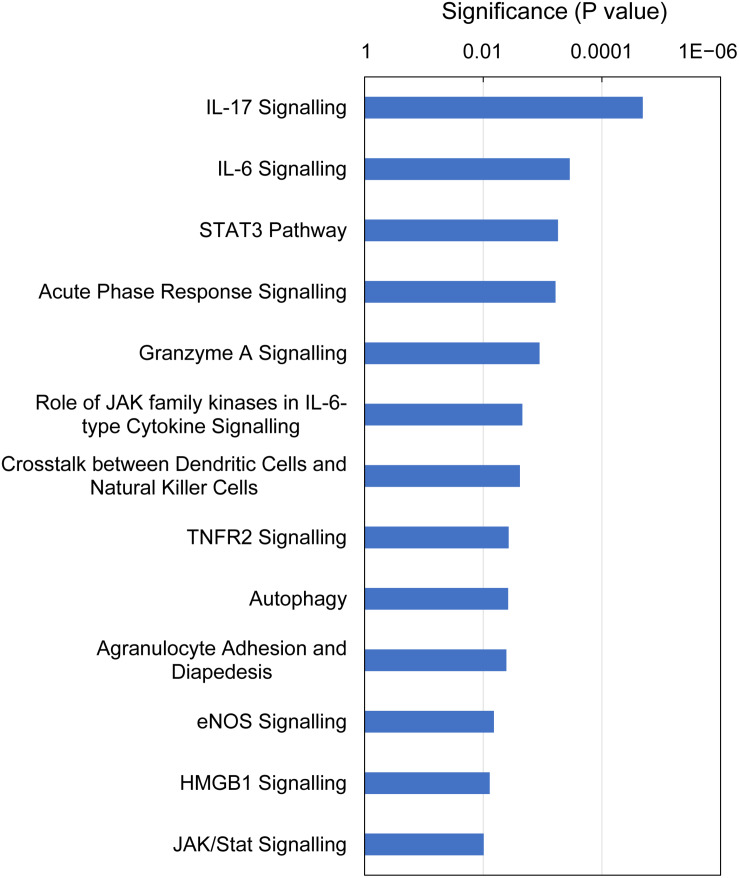
Top canonical pathways altered in Atlantic salmon gills with moderate histopathology (sites A and B) vs low histopathology (site C). The analysis was performed on 311 salmon genes mapped to 235 human orthologs, using Ingenuity Pathway Analysis (IPA) and *P*-value < 0.01. For details and list of corresponding genes see Supplementary Table 8.

IPA identified 13 canonical pathways that were altered in gills with moderate histopathology (sites A and B) vs low histopathology (site C) at *P*-value < 0.01, with contribution of 35 DEGs in total (Figure 6 and Supplementary Table 8). Most of these pathways are associated with 1) cellular immune response (IL-17 Signaling, IL-6 Signaling, Granzyme A Signaling, Crosstalk between Dendritic Cells and Natural Killer Cells, Agranulocyte Adhesion and Diapedesis and HMGB1 Signaling), 2) cytokine signaling (IL-17 Signaling, IL-6 Signaling, Acute Phase Response Signaling, Role of JAK family kinases in IL-6-type Cytokine Signaling, TNFR2 Signaling and HMGB1 Signaling) and (3) tissue damage and repair. The tissue damage and repair were evidenced by alterations of pathways related to cellular stress and injury (Autophagy and HMGB1 Signaling), apoptosis (TNFR2 Signaling and JAK/Stat Signaling), cellular growth (STAT3 Pathway and JAK/Stat Signaling) and proliferation and development (STAT3 Pathway and JAK/Stat Signaling).

**FIGURE 7:**
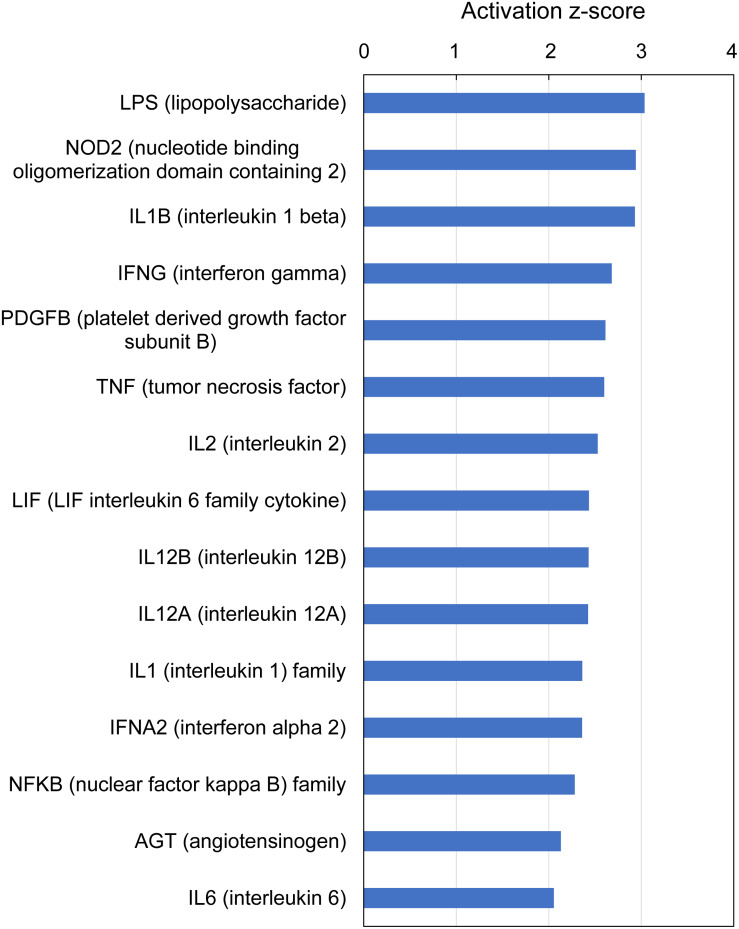
Top upstream regulators predicted from gene expression in Atlantic salmon gills with moderate histopathology (sites A and B) vs low histopathology (site C). The analysis was performed on 311 salmon genes mapped to 235 human orthologs, using Ingenuity Pathway Analysis (IPA). For details and list of corresponding genes see Supplementary Table 9.

IPA analysis of upstream regulators is based on prior knowledge of predictable effects between transcriptional regulators (e.g., transcription factors, cytokines, microRNAs, receptors, kinases, chemicals and drugs) and their target genes, stored in the Ingenuity Knowledge Base. When such analysis was performed on the gene expression profiles indicative of multifactorial gill disease, IPA identified 15 top upstream regulators associated with 101 of the 235 submitted DEGs, all highly significant (overlap *P*-value < 0.0001) and activated (*z*-score > 2) (Figure 7 and Supplementary Table 9). Among them were one endotoxin (LPS), one pattern recognition receptor (NOD2), 10 cytokines (IL1B, IFNG, TNF, IL2, LIF, IL12B, IL12A, IL1, IFNA2 and IL6), two growth factors (PDGF and AGT) and one transcription factor (NFKB).

**FIGURE 8:**
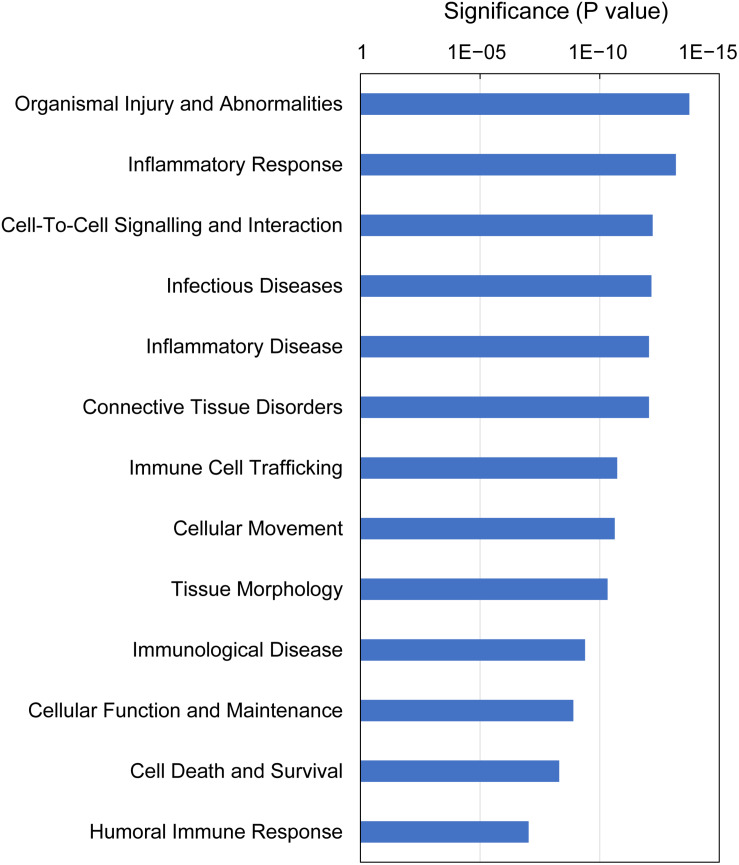
Top downstream effects predicted from gene expression in Atlantic salmon gills with moderate histopathology (sites A and B) vs low histopathology (site C). The analysis was performed on 311 salmon genes mapped to 235 human orthologs, using Ingenuity Pathway Analysis (IPA). For details and list of corresponding genes see Supplementary Table 10.

IPA analysis of downstream effects predicts potential outcomes from gene expression data, using the Ingenuity Knowledge Base of differential gene expression in varying disease and functional states. The predictions made for gills with moderate histopathology (sites A and B) vs low histopathology (site C) included 13 downstream disease/functions at *P*-value < 9.4E-08, with nearly all DEGs (230 of 235) contributing to the effects (Figure 8 and Supplementary Table 10). The dominant features of these predictions are (1) immune and inflammatory diseases (Inflammatory Response, Infectious Diseases, Inflammatory Disease, Immune Cell Trafficking, Immunological Disease and Humoral Immune Response), (2) tissue damage and repair (Organismal Injury and Abnormalities, Tissue Morphology, Connective Tissue Disorders, Cellular Movement, Cell Death and Survival, Cellular Function and Maintenance) and (3) intra-tissue communication (Cell-To-Cell Signaling and Interaction).

**FIGURE 9:**
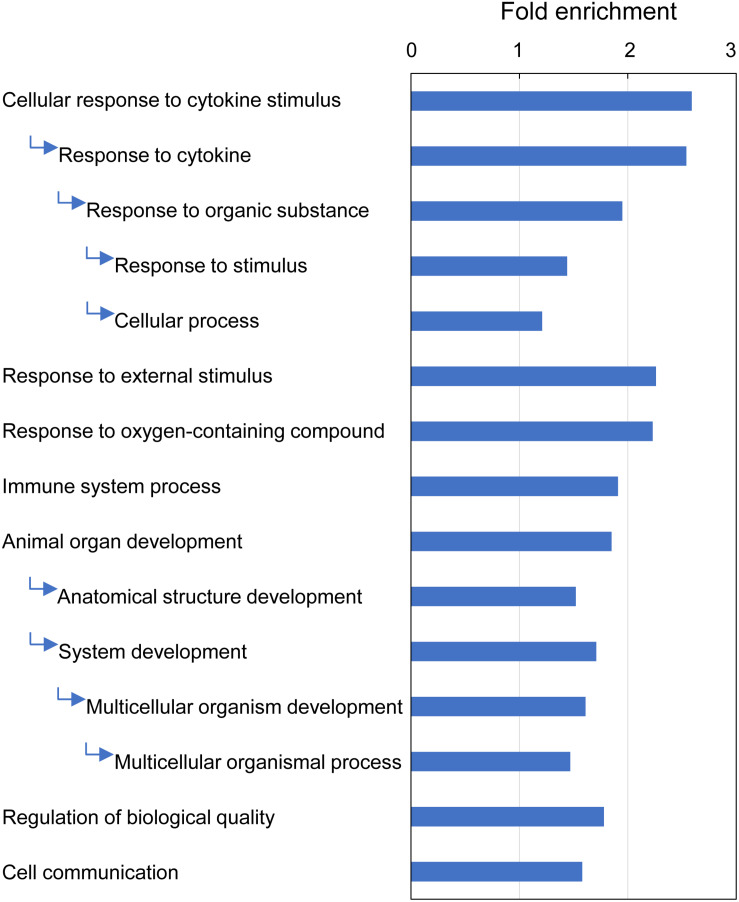
Top GO terms (Biological Process) associated with differentially expressed genes (DEGs) in Atlantic salmon gills with moderate histopathology (sites A and B) vs low histopathology (site C). The analysis was performed on 311 salmon genes mapped to 235 human orthologs, using PANTHER overrepresentation test and Bonferroni-corrected *P*-value < 0.05. The GO terms are sorted by the fold enrichment of the most specific categories, with their parent terms indented directly below. For details and list of corresponding genes see Supplementary Table 11.

GO enrichment analysis identified 15 GO terms (Biological Process) that were overrepresented with the majority of DEGs (209 of 235) associated with multifactorial gill inflammation, generating fold enrichments from 1.2 to 2.6 at Bonferroni-corrected *P*-value < 0.05 (Figure 9 and Supplementary Table 11). The enriched GO terms pointed towards increased (1) intra-tissue communication (Cellular response to cytokine stimulus and Cell communication), (2) presence of external stimuli (Response to external stimulus and Response to oxygen-containing compound), (3) activated immune response (Immune system process) and 4) ongoing tissue remodeling (Animal organ development and Regulation of biological quality).

### Top Genes Associated With Multifactorial Gill Disease

Top genes were defined here as the DEGs with the largest magnitude of change in expression (absolute Log_2_ FC > 2), thus including the protein-coding genes that were at least 4 times higher or 4 times lower expressed in gills with moderate histopathology (sites A and B) vs low histopathology (site C). The list of these top genes is presented in Table 6, with 43 upregulated salmon genes mapped to 25 unique human orthologs and 14 downregulated salmon genes mapped to 12 unique human orthologs.

## Discussion

Our study is the first to focus on the gill transcriptome of Atlantic salmon in a highly variable and complex environment of the marine production sites. We specifically chose to work on fish farmed in the open sea-based system (such as net pens), because (1) the use of net pens is currently the most common form of salmon production (Philis et al., 2019) and (2) in the open pens, only nets separate the fish from the environment, making them prone to developing gill pathologies but also assisting with the tissue recovery through high water exchange and oxygenation (Shinn et al., 2015; Nowak and Archibald, 2018). Farming in net pens is typically associated with the multifactorial gill diseases (PGD, PGI or CGD) rather than the single-cause gill pathologies, such as AGD (Herrero et al., 2018; Gjessing et al., 2019; Laurin et al., 2019). So far, the extensive transcriptomic profiling of the gill tissue has been performed only in salmon exposed to the controlled environment of the closed-system tanks, following a single-pathogen challenge to induce either AGD (Morrison et al., 2006; Wynne et al., 2008a; Bloecher et al., 2018; Boison et al., 2019; Robledo et al., 2019) or infectious salmon anemia (Valenzuela-Miranda et al., 2015). In most cases, fish were subjected to a single-dose challenge of the infectious organisms, with only few studies evaluating the effects of re-infection (Bloecher et al., 2018; Boison et al., 2019) or co-exposure to other infectious agents, such as hydroids (Bloecher et al., 2018) and *Yersinia ruckeri* (Valdenegro-Vega et al., 2015). To closely resemble the dynamics of multifactorial gill diseases observed in the production systems, fish need to be continuously exposed to a variety of infectious agents and environmental stressors that impact their health.

To evaluate the robustness and reliability of the PGD scores (gross morphology) in conveying information about gill health, we sampled salmon at three geographically distant locations in autumn and spring, without prior knowledge of the biotic and abiotic conditions of these sites. By doing this, we included in our data set the gill samples that were diverse in terms of origin, time of sampling, fish background and the overall environmental milieu (Table 1). The multi-site sampling approach to identify the common aspects of the multifactorial gill inflammation has been used before (Kvellestad et al., 2005; Steinum et al., 2010; Gjessing et al., 2019), but not in the context of the transcriptome analysis or underlying molecular mechanisms. Our study is the first to investigate the gill tissue from salmon at three different levels of organization: gross morphology (macroscopic examination), histopathology (microscopic examination) and whole transcriptome (gene expression) (Figure 1). We have focused on the gill arch with the highest PGD score per fish, as this arch may provide more accurate information about the general state of the gill health than the arch sampled randomly or the arch sampled because of its position, as practiced elsewhere (Wise et al., 2008; Steinum et al., 2010; Benedicenti et al., 2019; Gjessing et al., 2019). For better integration of the transcriptomic data with the gill gross morphology and histopathology, the tissue fragments used for the total RNA extraction aimed to represent the whole surface area of the gill rather than some specific areas of the tissue (Figure 1). This is in contrast to many gill gene expression (qPCR) studies, which tend to focus on either interbranchial lymphoid tissue or visible pathologies of the gill tissue, such as mucoid patches, hyperplastic lesions and lamellar fusions (Pennacchi et al., 2016; Marcos-López et al., 2018; Benedicenti et al., 2019).

### PGD Scores as Measures of Gill Health

Effective treatment of salmon gill diseases requires development of diagnostic and prognostic tools that are non-invasive and suitable for a frequent use in the rapidly changing marine environment (Herrero et al., 2018; Gjessing et al., 2019; Rozas-Serri, 2019). We have specifically focused on the applicability of the PGD scores (gross morphology) to reflect gill health and underlying pathology, as this scoring system is already in use as part of routine fish welfare indicator assessment of salmon health in seawater farms worldwide (Gill Health Initiative, 2015; Bloecher et al., 2018). By comparing 43 gill samples with low (1) and high (3) PGD scores across three marine production sites in Scotland, we found that these two groups of gill tissue classified as different at the macroscopic level (PGD 1 and PGD 3) were in fact indistinguishable at the level of whole-transcriptome gene expression (Figure 3A) and also indistinguishable at the level of microscopic histopathology (Figure 4A). Furthermore, we could not identify any single gene that was expressed differently between the two groups (Table 4). Our results strongly suggest that the changes in gross morphology were not supported by the changes in gene expression or histopathology. The lack of detectable transcriptomic and/or histopathological changes associated with the progression of the PGD scores questions the suitability of the gross scoring system as diagnostic and prognostic tools to monitor and control both existing and emerging gill diseases. Instead, the PGD scores are good proxies for monitoring changes in macroscopic gill surface area available for respiration, the knowledge of which may be essential for fish health management and husbandry practices in aquaculture settings.

**TABLE 6:** Top genes altered in Atlantic salmon gills with moderate histopathology (sites A and B) vs low histopathology (site C), based on FDR < 0.01 and absolute Log_2_ FC > 2.

Atlantic salmon genes				Human gene orthologs (HGNC identifiers)		
Gene ID	Transcript ID	Gene name	Log_2_ FC^1^	Symbol	Name	Log_2_ FC^2^
LOC106604507	XM_014199172.1	ribonuclease-like 3	4.6	ANG	angiogenin	4.6
LOC106598253	XM_014189304.1	ribonuclease-like 3	4.6	ANG	angiogenin	
LOC100196060	NM_001141089.1	angiogenin-1	4.5	ANG	angiogenin	
LOC100196525	NM_001141554.1	chymotrypsin-like	4.2	CTRL	chymotrypsin like	4.2
LOC106577309	XM_014155258.1	glutathione peroxidase 6-like	3.9	GPX2	glutathione peroxidase 2	3.9
LOC106561635	XM_014125761.1	ladderlectin-like	3.8	REG1B	regenerating family member 1 beta	3.8
LOC106577833	XM_014156195.1	interleukin-8-like	3.4	CXCL9	C-X-C motif chemokine ligand 9	3.4
LOC106601490	XM_014193713.1	RING finger protein 208-like	3.4	RNF152	ring finger protein 152	3.3
LOC106601491	XM_014193714.1	RING finger protein 186-like	3.2	RNF152	ring finger protein 152	
LOC106566533	XM_014134632.1	complement C1q tumor necrosis factor-related protein 3-like	3.2	C1QTNF3	C1q and TNF related 3	3.1
LOC106566537	XM_014134640.1	complement C1q tumor necrosis factor-related protein 3-like	3.2	C1QTNF3	C1q and TNF related 3	
LOC106566534	XM_014134633.1	complement C1q tumor necrosis factor-related protein 3-like	3.0	C1QTNF3	C1q and TNF related 3	
LOC106567034	XM_014135842.1	complement C1q-like protein 4	2.8	C1QL4	complement C1q like 4	2.8
LOC106573018	XM_014147615.1	carboxypeptidase A1-like	2.9	CPA1	carboxypeptidase A1	2.7
LOC106598577	XM_014189609.1	carboxypeptidase A1-like	2.8	CPA1	carboxypeptidase A1	
LOC100195857	NM_001140886.1	carboxypeptidase A1	2.6	CPA1	carboxypeptidase A1	
LOC106561558	XM_014125655.1	carboxypeptidase A1-like	2.3	CPA1	carboxypeptidase A1	
LOC100136358	XM_014214975.1	nitric oxide synthase 2	2.4	NOS2	nitric oxide synthase 2	2.4
LOC106594149	XM_014185514.1	C-type lectin domain family 4 member E-like	2.4	CLEC4E	C-type lectin domain family 4 member E	2.4
LOC106591222	XM_014182433.1	zymogen granule membrane protein 16-like	2.8	ZG16	zymogen granule protein 16	2.4
LOC106562680	XM_014127661.1	zymogen granule membrane protein 16-like	2.5	ZG16	zymogen granule protein 16	
LOC106562681	XM_014127663.1	zymogen granule membrane protein 16-like	2.4	ZG16	zymogen granule protein 16	
LOC106584756	XM_014170303.1	zymogen granule membrane protein 16-like	1.9	ZG16	zymogen granule protein 16	
LOC106567571	XM_014137044.1	H-2 class II histocompatibility antigen, A-K alpha chain-like	2.4	HLA-DPA1	major histocompatibility complex, class II, DP alpha 1	2.4
LOC106585685	XM_014172160.1	neurotrophic receptor tyrosine kinase 2	2.3	NTRK2	neurotrophic receptor tyrosine kinase 2	2.3
LOC100286614	NM_001146553.1	high choriolytic enzyme 2	2.7	ASTL	astacin like metalloendopeptidase	2.2
LOC106586984	XM_014174772.1	high choriolytic enzyme 1-like	2.7	ASTL	astacin like metalloendopeptidase	
LOC100195775	NM_001140804.1	high choriolytic enzyme 1	1.3	ASTL	astacin like metalloendopeptidase	
LOC106591797	XM_014183059.1	protein disulfide-isomerase-like	2.2	PDIA2	protein disulfide isomerase family A member 2	2.2
LOC106595494	XM_014186866.1	protein disulfide-isomerase A2-like	2.1	PDIA2	protein disulfide isomerase family A member 2	
LOC100196492	NM_001141521.2	cysteine dioxygenase type 1	2.1	CDO1	cysteine dioxygenase type 1	2.1
LOC100136458	NM_001123590.1	tumor necrosis factor alpha-2 precursor	2.1	LTA	lymphotoxin alpha	2.1
LOC106599048	XM_014190103.1	retinol-binding protein 1-like	2.1	RBP1	retinol binding protein 1	2.1
LOC106573692	XM_014148970.1	thyrotropin-releasing hormone receptor-like	2.1	TRHR	thyrotropin releasing hormone receptor	2.1
LOC106589989	XM_014180468.1	myosin-7-like	2.1	MYH7	myosin heavy chain 7	2.1
LOC106581492	XM_014163567.1	P2Y purinoceptor 13-like	2.1	P2RY12	purinergic receptor P2Y12	2.1
LOC106581219	XM_014163130.1	collagenase 3-like	2.5	MMP13	matrix metallopeptidase 1	2.1
LOC106613110	XM_014215041.1	collagenase 3-like	1.7	MMP13	matrix metallopeptidase 1	
LOC106581616	XM_014163747.1	aconitate decarboxylase 1	2.0	ACOD1	aconitate decarboxylase 1	2.0
LOC106609709	XM_014208680.1	complement C1q-like protein 2	2.3	C1QL2	complement C1q like 2	2.0
LOC106596487	XM_014187765.1	complement C1q-like protein 2	2.3	C1QL2	complement C1q like 2	
LOC106592632	XM_014183971.1	complement C1q-like protein 2	2.3	C1QL2	complement C1q like 2	
LOC106601034	XM_014192902.1	complement C1q-like protein 2	1.1	C1QL2	complement C1q like 2	
LOC106609915	XM_014208958.1	sialic acid synthase-like	−2.0	NANS	N-acetylneuraminate synthase	−2.1
LOC106594767	XM_014186155.1	sialic acid synthase-like	−2.1	NANS	N-acetylneuraminate synthase	
LOC106609889	XM_014208923.1	sialic acid synthase-like	−2.2	NANS	N-acetylneuraminate synthase	
LOC106562772	XM_014127771.1	neuronal acetylcholine receptor subunit alpha-7-like	−2.1	CHRNA7	cholinergic receptor nicotinic alpha 7 subunit	−2.1
LOC106566856	XM_014135351.1	succinate dehydrogenase [ubiquinone] iron-sulfur subunit, mitochondrial-like	−2.3	SDHB	succinate dehydrogenase complex iron sulfur subunit B	−2.3
LOC106603364	XM_014196939.1	sodium channel subunit beta-4-like	−2.4	SCN4B	sodium voltage-gated channel beta subunit 4	−2.4
LOC106594819	XM_014186204.1	corticosteroid 11-beta-dehydrogenase isozyme 2-like	−2.4	HSD11B2	hydroxysteroid 11-beta dehydrogenase 2	−2.4
LOC100194860	NM_001139889.1	Gamma-aminobutyric acid receptor subunit delta	−2.4	GABRD	gamma-aminobutyric acid type A receptor delta subunit	−2.4
LOC106607156	XM_014203794.1	heat-stable enterotoxin receptor-like	−2.4	GUCY2C	guanylate cyclase 2C	−2.4
LOC106597204	XM_014188425.1	pinopsin-like	−2.5	OPN3	opsin 3	−2.5
LOC106585409	XM_014171569.1	spermatid perinuclear RNA-binding protein-like	−2.6	STRBP	spermatid perinuclear RNA binding protein	−2.6
LOC106596671	XM_014187940.1	protein APCDD1-like	−2.9	APCDD1	APC down-regulated 1	−2.9
LOC106586762	XM_014174368.1	growth hormone-regulated TBC protein 1-A-like	−2.9	GRTP1	growth hormone regulated TBC protein 1	−2.9
LOC106606833	XM_014203242.1	uncharacterized LOC106606833	−3.1	MSLNL	mesothelin like	−3.1

*The Atlantic salmon genes are grouped by the common human ortholog (BLASTX, E-value < 0.00001, top hit) and sorted from the highest to the lowest Log_2_ FC associated with the HGNC identifier. ^1^Mean Log_2_ FC for A vs C and B vs C comparisons, as detailed in Supplementary Table 7; ^2^mean expression of salmon genes mapped to the same human ortholog.*

The PGD scoring system of salmon gill is based on the 6 macroscopic grades, from 0 (no visual pathology) to 5 (severe visual pathology) (Gill Health Initiative, 2015; Bloecher et al., 2018). It is important to realize that our comparison was done on the groups of tissue that differed only by two grades (PGD 1 and PGD 3) and were in the middle range of the spectrum, with a bias towards less damaged gills. Although our intention was to compare the transcriptome and histopathology of gill tissues with the full spectrum of PGD scores (e.g., PGD 0 and PGD 5) from the same farm location, fish with such a large range in gill gross morphology were not found during the sampling events, not only within the three production sites described in the current study (Table 2), but also at the four other locations (Scottish Sea Farms) visited by us in years 2017–2019 (R. Bickerdike and S. A. M. Martin, unpublished data). It is unusual for the salmon farmed in the sea to have all 8 gill arches scored as PGD 0 (apart from the first few weeks following the transfer from the freshwater facility), while the macroscopic gill damage classified as PGD 5 is very rare and would not be expected to be found until later in the production cycle, and as a result of a specific acute event or from cumulative significant environmental insults over a period of time (R. Bickerdike, personal communication).

Discrepancies between macroscopic and microscopic examination of the gill tissue have been reported by previous studies, mainly in the context of AGD (Pennacchi et al., 2016). For example, it has been shown that the AGD scoring system (gross morphology) may not be a reliable method of confirming the disease in cases of light severity of AGD (Clark and Nowak, 1999; Zilberg et al., 2001). This is because small AGD-associated lesions, which affect < 10 lamellae and are easy to detect under the microscope, are typically overlooked during the visual inspection of the gill (Adams et al., 2004). As a result, fish exposed to *Neoparamoeba perurans* and then classified as clear of the AGD symptoms during the macroscopic gill scoring for AGD may in fact need to be re-classified at the level of histopathological examination (Wynne et al., 2008b). Further complexity to the diagnostic problems is added when the gill disease is multifactorial (Wise et al., 2008; Gjessing et al., 2019; Noguera et al., 2019). More broadly, the poor diagnostic and prognostic value of the PGD scores demonstrated in our study is consistent with the limited applicability of the gross morphology to diagnose complex diseases in livestock and humans, which typically need extensive histopathology and molecular profiling to confirm and prognosticate (Hoffmann et al., 2009; Ahmed and Abedalthagafi, 2016; Mobadersany et al., 2018).

### Histopathology-Directed Analysis of Gill Transcriptome Between Sites

By sampling sea farmed Atlantic salmon at three production sites (A, B and C) in Scotland, we demonstrated that the gill samples from different sites had different histopathology (Figure 4B) and different transcriptomic profiles (Figure 3B), with 240 to 1360 genes identified as differentially expressed between the sites (Table 4). The drivers of this pronounced site-to-site variability in gill histopathology and transcriptome are unknown and may reflect the differences in (1) fish cohort, including their hatchery origin, past and present husbandry practices and overall health status, (2) local biotic conditions (e.g., diversity of pathogen), (3) local abiotic conditions (e.g., sea water temperature) and (4) interplay and interactions between all the above factors. Identification of these drivers requires more rigorous spatial and temporal sampling regime (Jokinen et al., 2012; Maestrini and Basso, 2018) and is beyond the scope of the current study.

The NMDS plots revealed that the gill samples from site C were different (i.e., clearly separated from sites A and B) not only at the level of their transcriptome (Figure 3B), but also in terms of their histopathology (Figure 4B). Because the NMDS ordination of the histopathological data is based on the same set of parameters (i.e., LH, LF, LO, CA, in, eg, cc, cd, ib, ch, pp and op) across all fish, lower NMDS1 values of gill samples from site C reflect lower scores of the contributing parameters (lower grade inflammation) than higher NMDS1 values of gill samples from sites A and B (Figure 4B, Supplementary Table 5, and Supplementary Figure 2). In contrast, the NMDS ordination of the transcriptomic data (Figure 3B) is based on the expression of 500 genes that are most divergent between each pair of fish (amounting to 903 sets of genes for all combinations of 43 fish in total) rather than being performed on the same set of genes across all fish (Robinson et al., 2010). Thus, the higher leading Log_2_ FC dim 1 values of gill samples from site C refer to their generally higher magnitude of transcriptomic changes rather than to the specific set of genes that separates them from sites A and B, with latter requiring more detailed analysis of the existing data to perform.

Because gill samples were more different between sites than within sites (Figures 3B, 4B), we performed the histopathology-directed analysis of gill transcriptome on all fish belonging to each site rather than on the individual fish within or between sites (Figure 5). Specifically, we classified all fish from site C as having gills with low histopathology, while all fish from site A and independently all fish from site B as having gills with moderate histopathology. Comparing the two independent sets of the moderately inflamed gill transcriptomes (A and B) with the low inflammation gill transcriptome (C), in the A vs C and B vs C contrasts, led to the identification of the common DEGs that are likely to constitute the gene expression patterns of multifactorial gill inflammation. Similar approaches utilizing independent sampling and site-to-site comparisons to establish common transcriptomic responses to a range of environmental factors have been successfully applied in studies ranging from aquatic toxicology (Oleksiak, 2008; Defo et al., 2018) to human medicine (Mueller et al., 2009; Rohart et al., 2017).

### Gene Expression Patterns of Multifactorial Gill Disease

Transcriptome profiling by RNA-seq has become a powerful tool for identification of genes and molecular pathways involved in the progression from health to disease in farmed fish (Sun et al., 2012; Martin and Król, 2017; Houston and Macqueen, 2019; Ronza et al., 2019). Combining RNA-seq assays with an independent sampling regime is especially important for understanding multifactorial diseases, because it allows for the potential identification of the common gene expression patterns of the disease that is essential for developing treatment and prevention strategies (Cookson et al., 2009; Bhuju et al., 2012). This approach has for example been used in the context of non-specific inflammatory diseases in fish intestine (reviewed in Martin et al., 2016). The present study is the first to focus on the transcriptomic patterns of multifactorial gill disease, a condition increasingly prevalent in salmon sea farms.

Transcriptomic comparison of the two independent sets of moderate histopathology gill samples (sites A and B) with the low histopathology gill samples (site C) led to the identification of 462 common DEGs in total, 311 of which were protein-coding transcripts mapped to 235 human orthologs (Table 5). The identification of these genes as part of the gene expression patterns of multifactorial gill disease was further supported by their uniform direction of changes in the expression levels (Figure 5). Subsequent functional analysis of these genes by IPA and GO pointed towards a range of immune responses as the most dominant feature of the moderate histopathology gills.

At the gene level, the immune responses were driven by pro-inflammatory cytokines (IL17F, CXCL9, CXCL10, CCL4L1 and TNF superfamily members: LTA and TNFSF14), cytokine receptors (TNFRSF1B, TNFRSF6B and CXCR1) and regulators of cytokine expression and signaling (C1QTNF3, SOCS1 and SOCS3), all of which were upregulated apart from TNFSF14 (Supplementary Table 7). The proteins encoded by IL17 family genes have been shown to stimulate the production of several other cytokines, including IL6 and IL8 in splenocytes of rainbow trout using recombinant IL17A/F2a (Monte et al., 2013) as well as IL1β, IL6, IL8 and TNF-α in head kidney leukocytes of grass carp using recombinant IL17A/F1 (Du et al., 2015). Furthermore, the emerging role of IL17 cytokines in gill mucosal immunity is supported by reports showing differential expression of these genes following the exposure to *Aeromonas hydrophila* in common carp (Dong et al., 2019) and after challenge with *Ichthyophthirius multifiliis* in rainbow trout (Syahputra et al., 2019). According to our BLASTX results, CXCL9 represents salmon IL8, whose high expression in our data set (Log_2_ FC = 3.4, Supplementary Table 7) is consistent with upregulation of IL17F (Zou and Secombes, 2016). The implication of IL8 in gill immune responses has been previously demonstrated in Atlantic cod (Caipang et al., 2010) and rainbow trout (Olsen et al., 2011; Santana et al., 2016). The protein encoded by LTA (salmon TNF-α2) mediates a large variety of inflammatory, immunostimulatory and antiviral responses in mammals (Upadhyay and Fu, 2013), showing induction at the level of gene expression in gills of Atlantic bluefin tuna during natural infection with Digenea (Pleić et al., 2015), but not in Atlantic salmon following the infection with *Neoparamoeba* spp. to induce AGD (Morrison et al., 2007). Cytokines were also overwhelmingly present in our functional analysis of gene expression patterns in the moderately inflamed gills. Specifically, 6 of the top 13 IPA canonical pathways were related to cytokine signaling (Figure 6 and Supplementary Table 8) and 10 of the top 15 IPA upstream regulators were predicted to be cytokines (Figure 7 and Supplementary Table 9). The presence of cytokines among DEGs and also as upstream regulators is consistent with a cytokine signaling cascade, during which one cytokine stimulates its target cells to produce another cytokines (Tisoncik et al., 2012). As a result of cytokine presence among DEGs, 6 of the top 13 IPA downstream effects were predicted to be immunological and inflammatory diseases (Figure 8 and Supplementary Table 10). Altogether, our results identified cytokine-driven immune response as a hallmark of multifactorial gill disease.

Besides the immune response, the multifactorial gill disease from sites A and B had a common transcriptomic profile indicative of tissue damage and repair. Part of the tissue damage could be inflicted by high levels of nitric oxide (NO), suggested by upregulation of NOS2 (Log_2_ FC = 2.4, Supplementary Table 7). This gene encodes a NO synthase that is inducible by a combination of lipopolysaccharide (LPS) and certain cytokines (Okamoto et al., 2004). Although NO is produced as a first-line defense against invading pathogens, its strong cytotoxic effects may also damage the tissue of the host (Abramson et al., 2001). Furthermore, high levels of NO may contribute to oxidative stress through production of reactive oxygen species (ROS) (Girouard et al., 2009), which could potentially explain the upregulation of GPX2 in our data set (Log_2_ FC = 3.9, Supplementary Table 7). The protein encoded by this gene belongs to the glutathione peroxidase family, members of which catalyze the reduction of organic hydroperoxides and hydrogen peroxide (H_2_O_2_) by glutathione, and thereby protect cells against oxidative damage (Brigelius-Flohé and Maiorino, 2013). The potential role of GPX2 in the antioxidant defense against increased ROS at gill mucosal surfaces has been recently discussed in the context of exposing Atlantic salmon to peracetic acid (Soleng et al., 2019). It is well established that the repair of inflamed tissue requires efficient removal of damaged cells through controlled cell death (apoptosis) and concurrent cell proliferation to regenerate damaged structures and build up lost tissue, with both processes closely linked to the activity of cysteine-dependent aspartate-directed proteases (caspases) (Fogarty and Bergmann, 2017). In our study, both CASP14 (salmon caspase-14-like) and other genes associated with apoptosis and autophagy (RNF152, CTSL, CTSG, CTSV, RAB32 and TGM2) were upregulated, pointing towards ongoing tissue repair in the moderately inflamed gills (Supplementary Table 7). The increased activity of caspases during gill inflammation has been previously documented in rainbow trout challenged with *Aeromonas salmonicida* (Rojas et al., 2015) and grass carp after infection with *Flavobacterium columnare* (Chen et al., 2019). Because of the complexity and size of gill vasculature, the repair of the gill tissue requires extensive vasculogenesis (formation of new blood vessels from vascular precursor cells), angiogenesis (process of outgrowing vessels from the existing vasculature) and arteriogenesis (remodeling of arteries where collateral arterial anastomoses undergo abluminal expansion) (Shi et al., 2017). One of the most potent mediators of new blood vessel formation is angiogenin (Hoang and Raines, 2017), represented in our data set by ANG (salmon angiogenin-1 and ribonuclease-like 3), which was the gene with the highest level of upregulation (Log_2_ FC = 4.6, fold change ∼24) in the moderate histopathology vs low histopathology gills (Table 6 and Supplementary Table 7). This result is consistent with the earlier study conducted by Valdenegro-Vega et al. (2014), showing increased protein levels of angiogenin (fold change ∼12) in the gills of Atlantic salmon following four successive infections with *Neoparamoeba perurans*. Among other genes involved in angiogenesis were ADM, BMP10 and VCAN, all upregulated in the moderately inflamed gills (Supplementary Table 7). Overall, 5 of top 13 IPA canonical pathways (Figure 6 and Supplementary Table 8) and 5 of top 13 IPA downstream effects (Figure 8 and Supplementary Table 10) were associated with cell death and proliferation related to tissue damage and repair, highlighting their importance in multifactorial gill disease.

### Conclusion and Future Directions

The gill is central for understanding the impacts of climate change on fish health. Recent increases in gill pathologies in sea farmed Atlantic salmon highlight the need to establish the molecular basis of multifactorial gill disease (frequently referred to as PGD, PGI or CGD) to improve diagnosis and preventive measures of this condition. To ensure the multifactorial etiology of gill disease, we sampled Atlantic salmon from three different production sites in Scotland and then examined the gill tissue at three different levels of organization: gross morphology with the use of PGD scores (macroscopic examination), whole transcriptome (gene expression by RNA-seq) and histopathology (microscopic examination). By exploring the association between gill transcriptome and gill gross morphology (PGD scores), we found that the PGD scores were less effective in providing a graded assessment of gill health status than expected, and they did not convey any information about the underlying pathology and/or tissue deterioration. In contrast, integration of the gill RNA-seq data with the gill histopathology enabled us to identify common gene expression patterns associated with multifactorial gill disease. We demonstrated that the gene expression patterns associated with multifactorial gill disease were dominated by two processes: a range of immune responses driven by pro-inflammatory cytokines and the events associated with tissue damage and repair, driven by caspases and angiogenin.

Previous studies of the gill inflammation have typically focused on single-cause pathologies (such as AGD), in the experiments performed on fish exposed to the controlled environment of the closed-system tanks. Although these studies are very important from a mechanistic point of view of specific pathologies, they may have limited relevance for the fish facing a changing environment at farm locations, e.g., rising sea water temperature, deoxygenation and surges of existing and emerging pathogens with the complexity of co-infections, coupled with husbandry practices. Performing more transcriptomic studies in the field rather than in the lab would benefit both academia and industry. The multi-site approach is also important because in this study, we demonstrated large and significant effects of sampling site (sensu lato) on gill transcriptome and histopathology. The drivers of this site-to-site variability are presently unknown and require more specific sampling regime.

Our histopathology-directed analysis of gill transcriptomes identified in total 462 genes that we claim to constitute the gene expression profile of multifactorial gill inflammation, including 354 protein-coding genes, 35 immunoglobulin gene segments, 15 pseudogenes and 58 non-coding RNAs. It is, however, important to remember that our analysis was based on the gill samples from three production sites in one specific part of the world (coastal waters of Scotland), with sampling events covering October, November and March. Substantial amount of work is therefore needed to test the association of these genes with multifactorial gill diseases at different times of year in Scotland and worldwide. The diagnostic and therapeutic value of these transcripts is currently unknown and require further studies.

## Data Availability Statement

The RNA-seq data have been deposited in the ArrayExpress repository (http://www.ebi.ac.uk/arrayexpress/) under accession number E-MTAB-8855. The R and Python scripts are available on request to AD and SS, respectively.

## Ethics Statement

Ethical review and approval was not required for the animal study because the sampling of fish was carried out under established protocols for routine health assessments in accordance with RSPCA Assured Welfare Standards for farmed Atlantic salmon and under supervision of the company veterinarian.

## Author Contributions

SM, AD, EK, VV, KG, and RB conceived and designed the study, interpreted results, and gave final approval of the manuscript. All authors (apart from SS and AD) were involved in sampling. EK extracted RNA, performed IPA analysis, and drafted the manuscript with AD, who oversaw bioinformatics and statistical analysis. SS mapped salmon genes to human orthologs to enable functional analysis of gene expression. PN performed histopathological examination of samples, generated scores, and interpreted results. EC provided support in data exploration. All authors discussed and commented on the manuscript.

## Conflict of Interest

KG and VV were employed by the company BioMar AS. RB was employed by the company Scottish Sea Farms (SSF). The remaining authors declare that the research was conducted in the absence of any commercial or financial relationships that could be construed as a potential conflict of interest.

## Footnotes

1 http://www.ebi.ac.uk/arrayexpress/

2 https://www.ensembl.org/Homo_sapiens/Info/Index
