# Supplementary material for: Integration of Transcriptome, Gross Morphology and Histopathology in the Gill of Sea Farmed Atlantic Salmon (Salmo salar): Lessons From Multi-Site Sampling
Source: Front Genet. 2020 Jun 19;11:610. doi: 10.3389/fgene.2020.00610 (PMC7316992; doi:10.3389/fgene.2020.00610)
Supplement: Supplementary file 1 [file Data_Sheet_1.PDF]

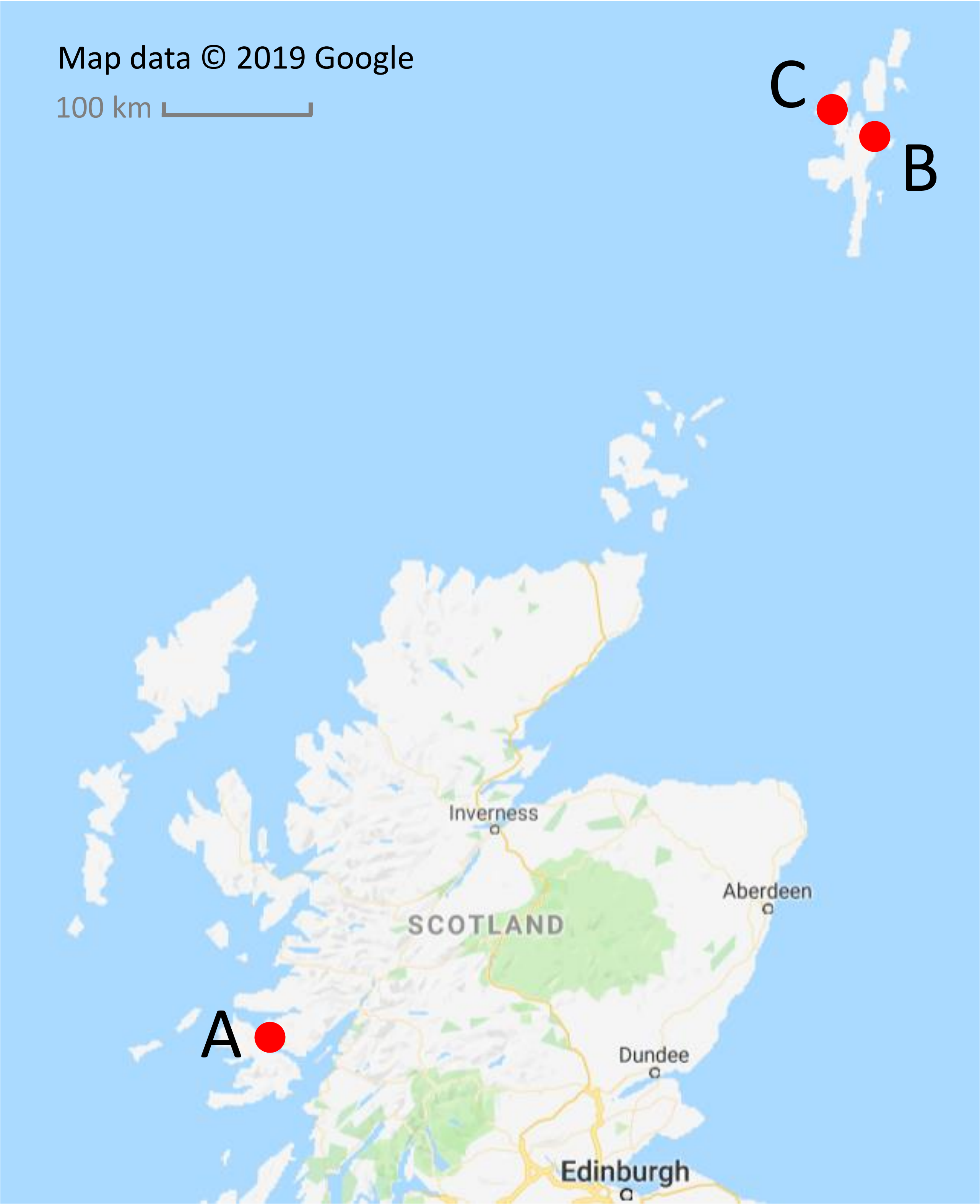

Supplementary Figure 1. Localization of sampling sites (Scottish Sea Farms): A (Isle of Mull), B (Shetland) and C (Shetland). Map

data were obtained from Google Maps (https://www.google.com/maps/).
