# Supplementary material for: Integration of Transcriptome, Gross Morphology and Histopathology in the Gill of Sea Farmed Atlantic Salmon (Salmo salar): Lessons From Multi-Site Sampling
Source: Front Genet. 2020 Jun 19;11:610. doi: 10.3389/fgene.2020.00610 (PMC7316992; doi:10.3389/fgene.2020.00610)
Supplement: Supplementary file 2 [file Data_Sheet_2.PDF]

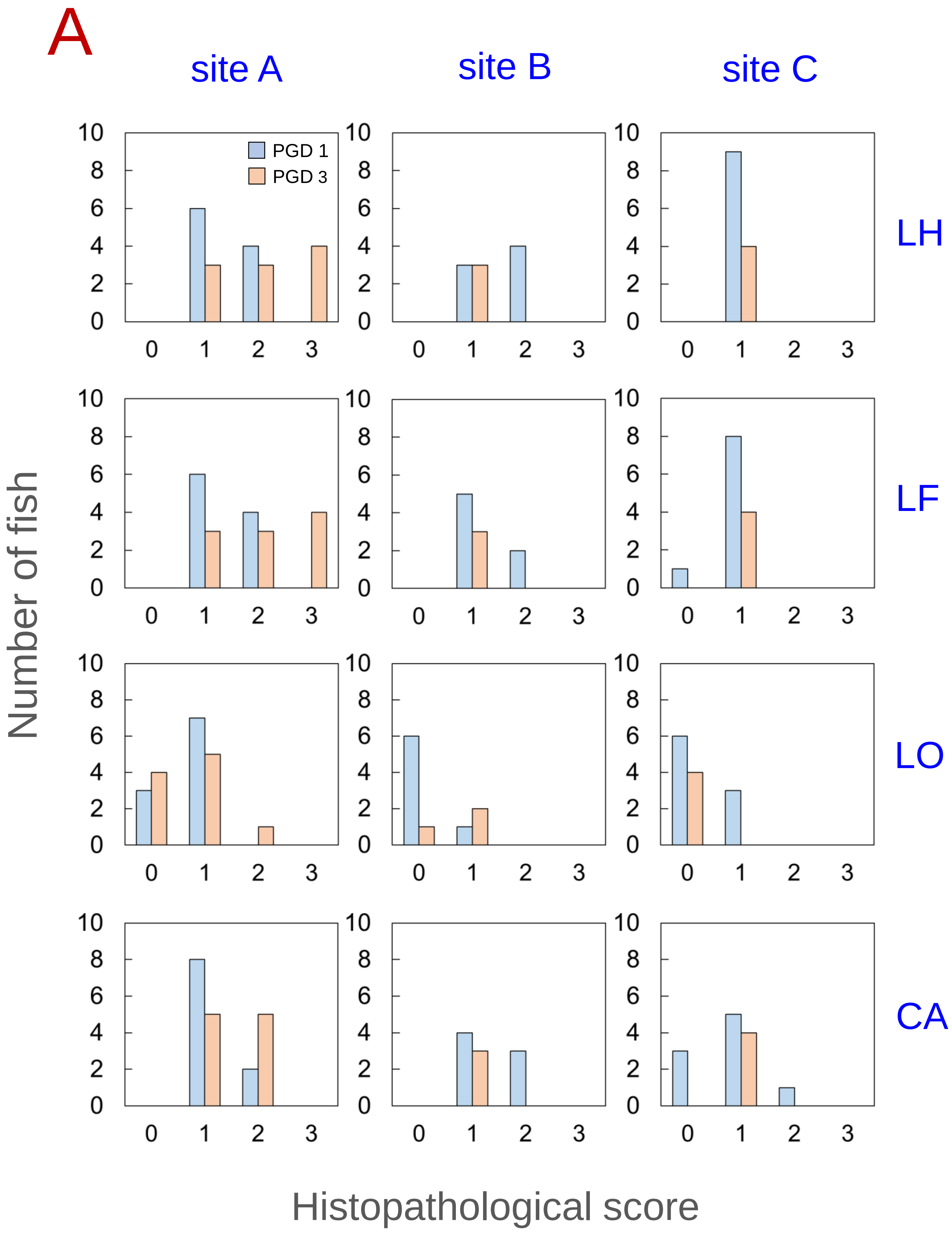
A
site A
site B
site C
PGD 1
PGD 3
LH
LF
LO
CA
Number of fish
Histopathological score

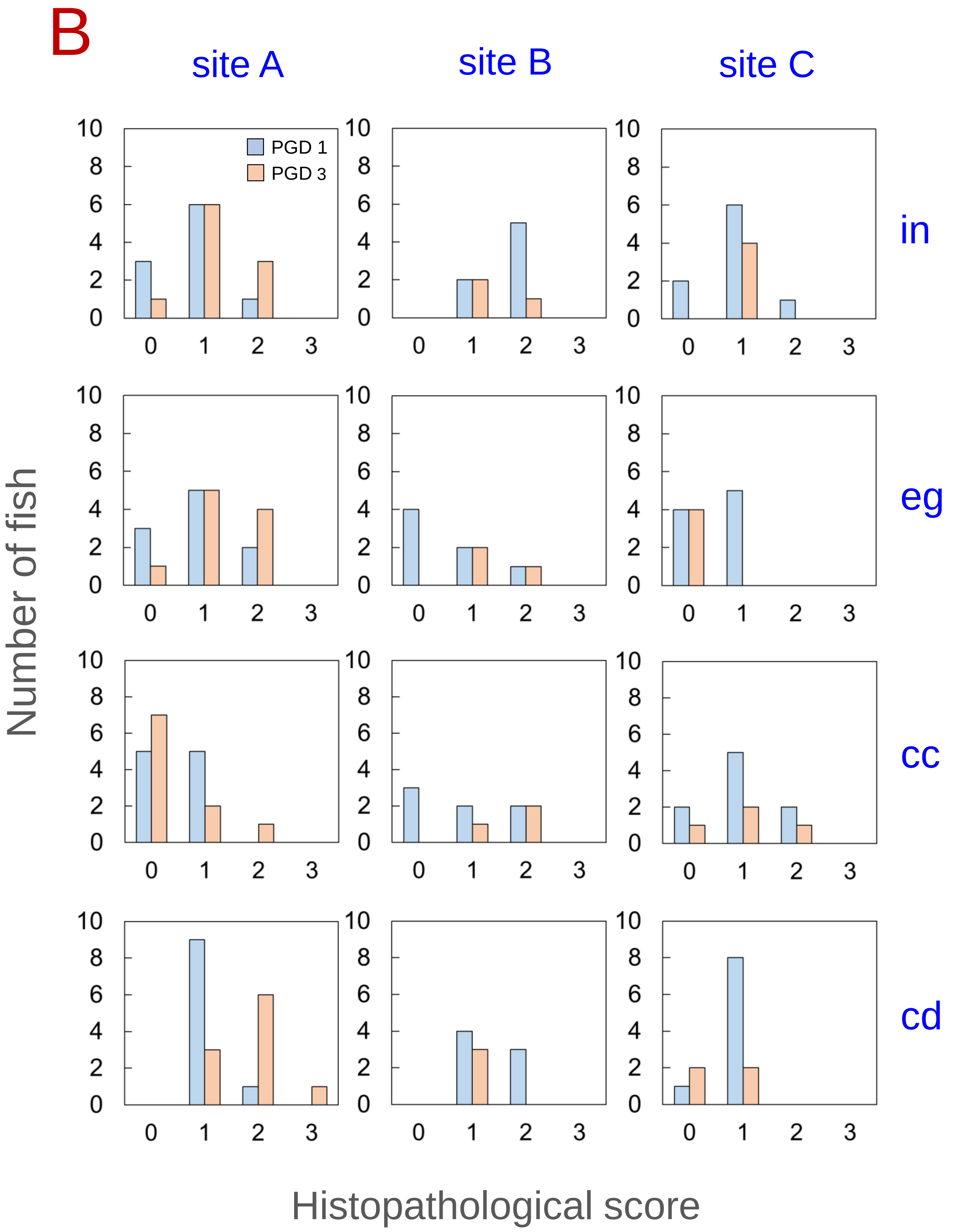
B
site A
site B
site C
PGD 1
PGD 3
in
eg
cc
cd
Number of fish
Histopathological score

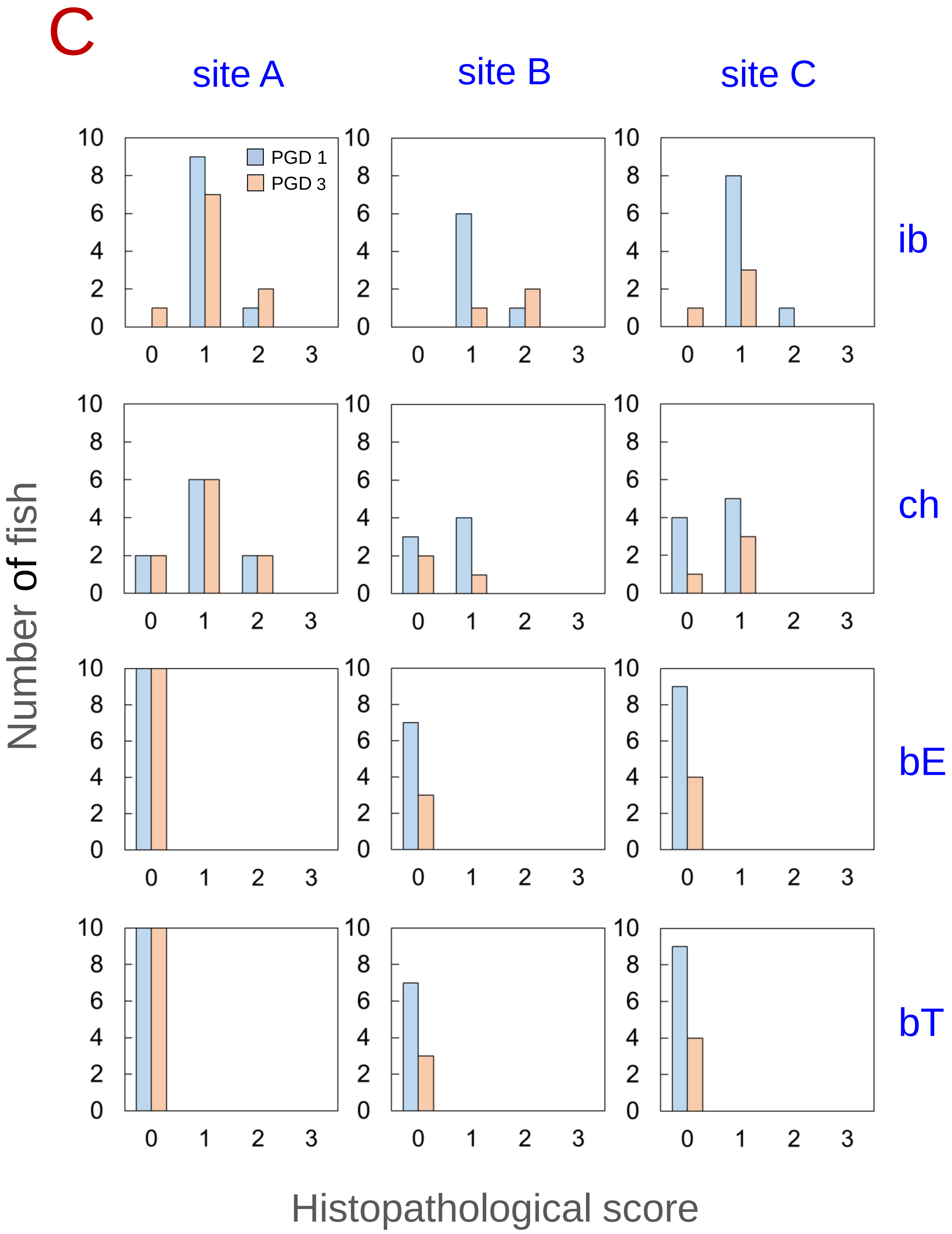
C
site A
site B
site C
PGD 1
PGD 3
ib
ch
bE
bT
Number of fish
Histopathological score
0
1
2
3
10
8
6
4
2
0

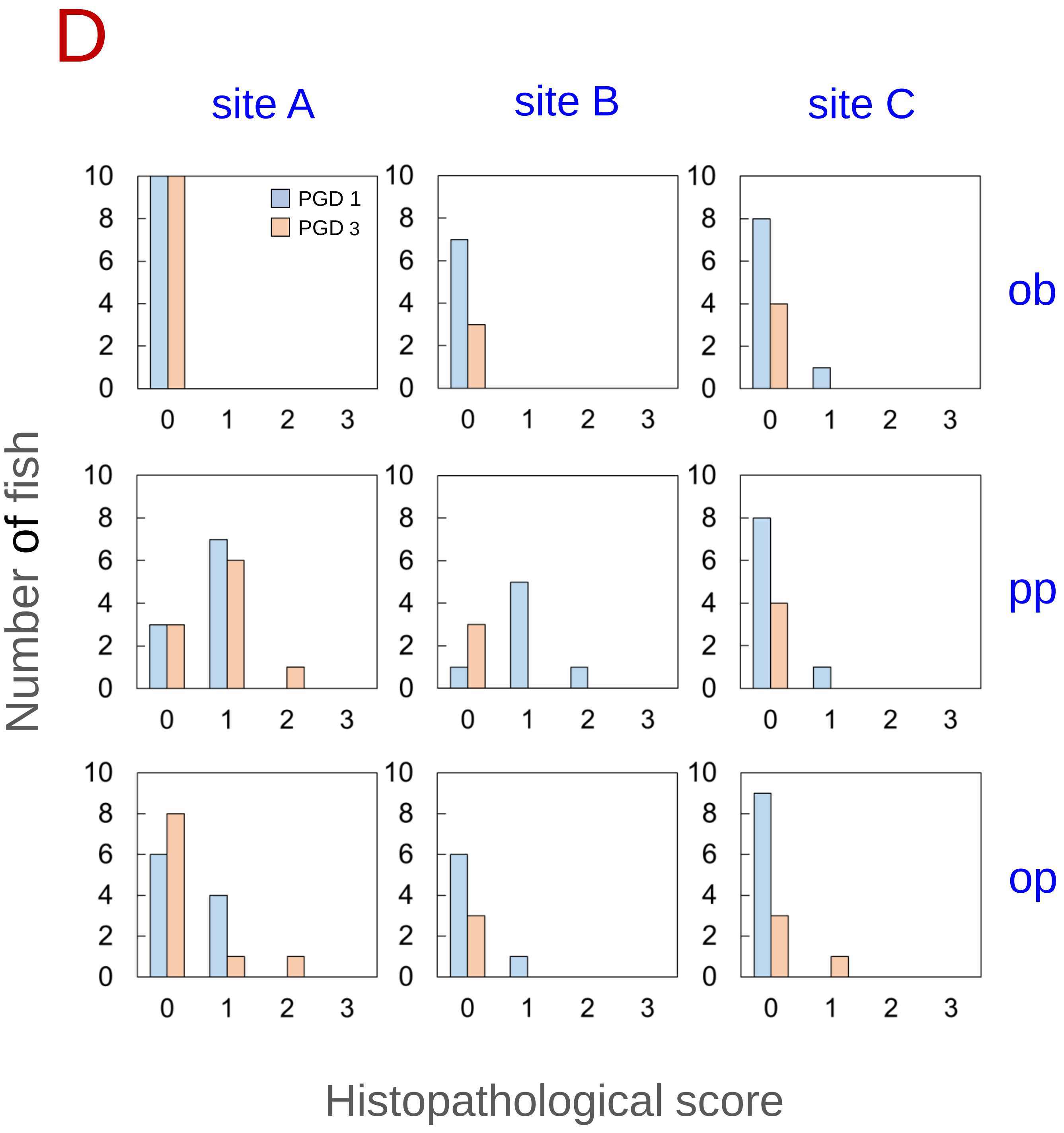

Supplementary Figure 2. Number of fish with histopathological scores per group (PGD 1 and PGD 3) and site (A, B and C) for (**A**) gill lamellar hyperplasia (LH), lamellar fusion (LF) lamellar

oedema (LO) and cellular anomalies (CA), (**B**) gill inflammation (in), eosinophilic granular cells (eg), chloride cells (cc) and circulatory disturbances (cd), (**C**) gill interlamellar blood (ib), cellular hypertrophy (ch), *Epitheliocystis*-like bacteria (bE) and *Tenacibaculum*-like bacteria (bT) and (**D**) presence in the gill of other bacteria (ob), protist parasites (pp) and other parasites (op). For details, see Table 3 and Supplementary Table 5.
